# Supplementary material for: Evaluating the effect of the SMART intervention in people with recently diagnosed breast cancer who are being treated at a public tertiary hospital in Australia: protocol and statistical analysis plan for a single-blinded, single centre randomised controlled trial
Source: PLoS One. 2026 Jan 30;21(1):e0341423. doi: 10.1371/journal.pone.0341423 (PMC12857944; doi:10.1371/journal.pone.0341423)
Supplement: S1 File — (DOCX) [file pone.0341423.s001.docx]

***Supplementary Table 1: Trial registration data***

| **Data category** | **Information** |
| --- | --- |
| Primary registry and trial identifying number | ACTRN12623001168640p |
| Date of registration in primary registry | 10/11/2023 |
| Secondary identifying numbers | S01/2023 |
| Source(s) of monetary or material support | Royal Perth Hospital Research Foundation |
| Primary sponsor | Royal Perth Hospital Physiotherapy Department |
| Secondary sponsor(s) | Curtin University |
| Contact for public queries | Kylie Hill: k.hill@curtin.edu.au |
| Contact for scientific queries | Kylie Hill: k.hill@curtin.edu.au |
| Public title | The effect of exercise intervention for adults undergoing treatment for breast cancer |
| Scientific title | The effect of exercise intervention on quality of life for adults undergoing treatment for breast cancer using a Self-Determined, Monitored, Adaptable Rehabilitation with Telehealth support (SMART) exercise intervention |
| Countries of recruitment | Australia |
| Health condition(s) or problem(s) studied | Breast Cancer |
| Intervention(s) | Exercise intervention called the SMART (Self-determined, monitored, adaptable, rehabilitation with telehealth support) Intervention. |
| Key inclusion and exclusion criteria | Adults will be eligible to participate if they meet the following criteria:   1. Adult with newly diagnosed breast cancer 2. Planned treatment includes neo-adjuvant or adjuvant chemotherapy AND/OR endocrine therapy   Adults will not be eligible to participate if the meet any of the following criterial   1. Cognitive impairment or are unable to speak or read written English 2. Unable to access internet/phone app 3. Living in supported residential care 4. Any other co-morbidities where exercise is contra-indicated, at the discretion of the treating specialist |
| Study type | Interventional  Randomised controlled trial |
| Date of first enrolment | 17/06/2025 |
| Target sample size | 260 |
| Recruitment status | Recruiting |
| Primary outcome(s) | Health releated quality of life using the EORTC Quality of Life C30 questionnaire with the breast cancer specific BR45 module and the EQ5D5L |
| Key secondary outcomes | Absenteeism from work  Chemotherapy completion rates  Health care utilisation  Capability, opportunity, and motivation to exercise  Impairment in function  General self-control  Physical exercise-related barrier self-efficacy  Habitual tendencies  Mood  Physical exercise-related task self-efficacy  Muscle strength  Walking tolerance  Body composition  Physical activity |
