# Supplementary material for: Evaluating the effect of the SMART intervention in people with recently diagnosed breast cancer who are being treated at a public tertiary hospital in Australia: protocol and statistical analysis plan for a single-blinded, single centre randomised controlled trial
Source: PLoS One. 2026 Jan 30;21(1):e0341423. doi: 10.1371/journal.pone.0341423 (PMC12857944; doi:10.1371/journal.pone.0341423)
Supplement: S2 File — (PDF) [file pone.0341423.s002.pdf]

WA HEALTH RESEARCH PROTOCOL

The SMART exercise program for people recently diagnosed with breast cancer

Table of Contents

1 Trial Details 3
Trial Summary 3
2 Rationale / Background 3
Background summary 3
Intervention 4
3 Trial Aims / Objectives / Hypotheses 5
4 Trial Design 6
Study Endpoints 6
Study Design 6
Bias 6
Blinding and Randomisation 6
Intervention/Product Description 7
Product Accountability Procedures 9
Trial Duration/Schedule 9
Trial Termination 9
Data identification 9
5 Source and Selection of Participants 10
Source of Participants 10
Participant inclusion criteria 10
Participant exclusion criteria 10
Participant withdrawal criteria 10
6 Treatment of Participants 10
Description and justification for treatments, interventions or methods to be utilised 11
7 Assessment of Efficacy 11
Efficacy assessment 13
8 Assessment of Safety 15
Risks and benefits 15
Safety 15
Data and Safety Monitoring Board 15
Adverse event reporting 15
Follow-up of Adverse Events 15
9 Data Management, Statistical Analysis and Record Keeping 15
Statistics and Interim Analysis 16
Sample Size 17
Study Power and Significance 17
Statistical plan deviations: 17
Selection of participants for analyses: 17
Data management 17
Procedures for missing, unused and spurious data: 18
10 Monitoring / Audit 18
Monitoring, Audit and Regulatory Inspections Statement 18
Procedures for monitoring and auditing 18
11 Quality Control and Quality Assurance 18
Compliance statement. 18
Quality control 18
12 Ethics 18
13 Budget, Financing, Indemnity and Insurance 18
14 Publication 19
15 References 21
16 Appendices 23
1. Investigator's Brochure ....
2. Proposed budget ....
3. Staff and resource requirements ....

1 Trial Details

**Protocol/Clinical Trial Title:**	The *SMART* exercise program for people recently diagnosed with breast cancer		
**Protocol Number (Version and Date):**	Version 3 (20th August, 2024)		
**Amendment (Number and Date):**	Amendment 2 (25th July, 2024)		
**Trial Start Date:**	1/1/2024	**Trial Finish Date:**	04/10/2027
**Coordinating Principal Investigator Name:**	Kylie Hill		
**Coordinating Principal Investigator Contact Details:**	K.Hill@curtin.edu.au; 0411144867		
**Sponsor Name (if applicable):**	N/A		
**Laboratory Name (if applicable):**	N/A		

Trial Summary

Breast cancer (BC) is a common malignant disease in women worldwide. Most women with BC undergo complex treatment regimens including surgery to remove the tumour and affected lymph nodes, as well as chemotherapy, radiation and/or endocrine therapy. Treatment for BC results in many side effects that compromise physical and emotional health. The time-consuming demands of the treatment itself together with the treatment side effects result in time away from work and/or other meaningful life activities.

There is emerging evidence that exercise interventions undertaken during or after completion of cancer treatment optimises physical function, emotional health and reduces fatigue. Australian and international cancer organisations now advocate that individuals should participate in regular exercise while undergoing their BC treatment. Although usual care pathways at the Royal Perth Hospital (RPH) Breast Clinic physiotherapy service involves education of the importance of exercise, it is possible that a more structured, individualised and supervised approach to exercise prescription would produce greater benefit in this population of patients, including improved physical activity maintenance after completion of the program.

Our study will explore the effect of an experimental exercise program (known as the *SMART* program; [**S**elf-determined **M**onitored **A**daptable **R**ehabilitation with **T**elehealth support]) in adults accessing the Breast Clinic at RPH. The experimental program will be tailored to each person and provide supervised exercise sessions either in person or via telehealth in addition to regular behaviour change interventions. The primary outcome will be disease-specific health-related quality of life. Secondary outcomes will include peripheral muscle strength, exercise tolerance, fatigue, chemotherapy completion rates, healthcare utilisation, psychological determinants of behaviour change and mood.

2 Rationale / Background

Background summary

Breast cancer is a common disease, affecting 1 in 8 women over the course of their life (Cancer Australia 2023). In 2020, the worldwide incidence of BC was 2.26 million (Ferlay et al., 2020). Fortunately, survival rates from BC have improved dramatically such that currently, 9 out of 10 women remain in remission at 5 years following initial diagnosis (Giaquinto et al., 2022). Treatment for BC often includes surgical excision of the tumour and affected lymph nodes, chemotherapy, endocrine therapy and/or radiotherapy. Side effects of BC treatments include but are not limited to loss of skeletal muscle mass, reduced cardiovascular fitness, fatigue, pain, depressive symptoms and gastrointestinal symptoms (Bland 2019, Collins 2018, Feliciano 2017, Mijwel 2018). These side effects can persist for many years and serve to reduce health-related quality of life and physical function (Brown 2012). Addressing quality of life following treatment is of paramount importance, particularly with reports of

worsening quality of life 2-years after diagnosis (Ferreira 2019). Implementation of therapies and/or strategies to address these sequalae is urgently needed.

Encouragement to exercise has become part of usual care during and following completion of BC treatment. Data from several systematic reviews investigating the effects of exercise demonstrate positive effects on overall health-related quality of life (Mishra 2012; Zhang 2019), pain (Plinsinga 2022), fatigue (Meneses- Echavez 2015), physical function (Ficarra 2021) and mood (Salam 2022). Further, exercise interventions have been shown to reduce declines in physical fitness (Ficarra 2021) and improve chemotherapy completion rates (van Waart 2015). Importantly, exercise post-diagnosis significantly reduces BC-related mortality (Salam 2022). Despite this growing body of literature, effect sizes are modest and wide confidence intervals published across systematic reviews challenge our confidence in that effects will be clinically meaningful. This highlights the need for ongoing research to optimize outcomes from exercise interventions.

Nevertheless, National and International organisations e.g., the Clinical Oncology Society of Australia (COSA) routinely recommend that following any cancer diagnosis, individuals should complete at least 150 minutes per week of moderate intensity aerobic exercise or 75 minutes per week of vigorous intensity aerobic exercise, as well as two resistance training sessions each week (COSA 2018, World Cancer Research Fund, 2018). Exercise during cancer treatments has been widely studied and reported as a safe intervention for individuals with cancer (Cormie 2018). However, despite the clear clinical guidelines and safety reports, approximately 80% of women with BC do not meet these exercise recommendations and 23% reduce their usual activity level during cancer treatment (Gildea 2023). Systematic reviews suggest that even if supervised exercise programs successfully increase physical activity levels, these physical activity levels were maintained in only four out of ten trials (Weemaes et al., 2023). Women with BC commonly report that transitioning from supervised to self-managed exercise is one of the most significant barriers to maintaining physical activity (Ferri et al., 2021). Hence, there is a clear need to develop strategies to improve adherence to participation in exercise in this population.

Engaging in regular exercise during chemotherapy is arguably more challenging relative to other phases of the treatment trajectory due to common and debilitating physical side effects, such as fatigue, nausea and cancer related pain (Kirkham 2014). Such side effects from chemotherapy are the most commonly reported barrier to adherence to exercise during chemotherapy treatment for women with breast cancer (Kirkham 2014). Other cited barriers include inconvenient exercise locations, disease stage and poor baseline strength or fitness (Courneya 2008, Gildea 2023). Currently, as part of usual care, people known to the Breast Clinic at RPH are seen by a physiotherapist who provides education regarding the importance of exercise during their cancer treatment. Patients are provided with a pamphlet and encouraged to engage in appropriate off-site exercise groups. It is understood that uptake of exercise is suboptimal. This study will explore the effect of an experimental exercise program, known as the is *SMART* program. This intervention has been co-designed, along with people who have BC, to overcome many of the known barriers to exercising during their cancer treatment. The SMART program will include individualised exercise prescription, monitoring of adherence, goal setting, strategies to optimise social support, action and coping planning and regular ‘coaching’ support to guide individuals through their exercise intervention. Importantly, this intervention will be available to people known to RPH, but who cannot access RPH due to transport problems and because they live outside Perth (i.e. rural and remote regions).

Intervention

A randomised controlled trial will be conducted. The experimental group will receive the *SMART* exercise program. To optimise adherence, participants in the SMART exercise program will be offered weekly one-on-one sessions with a senior physiotherapist for exercise prescription, monitoring or exercise uptake and potential treatment side effects, and to facilitate behaviour change. The target behaviour of the SMART program is to assist participants to build up to or maintain 150 minutes per week of moderate intensity aerobic exercise (or 75 minutes per week of vigorous intensity exercise) as well as two resistance training sessions per week. This target has been informed by the 2019 American College of Sports Medicine (ACSM) guidelines for exercise and cancer. Specific details of the SMART program have been described in Section 4 (under Intervention / Product Description).

The principles underpinning the SMART exercise program are that it will be:

Self-determined: Participants will be in the driver's seat regarding the establishment of weekly goals. They can choose to engage in exercises they enjoy (walking, swimming, cycling, dance etc) at the time of day that suits them best. Due to expected variation in baseline fitness levels and exercise preferences, the program will be individualised to each participant.

Monitored: The monitoring of adherence and treatment fidelity will occur through exercise diaries, monitoring of heart rate during the supervised and unsupervised exercise sessions and regular questionnaire data to monitor potential side effects relating to BC treatment and exercise.

Adaptable: The participant can choose the days/times they want to exercise and whether the weekly (supervised) session is completed at RPH, or via telehealth. In addition, participants will be briefed to periodise exercise routines according to potential BC treatment side effects (e.g. fatigue, pain, nausea) reported used the above monitoring strategies. Specifically, exercise programs will be adapted if the participant reports any symptoms (e.g. if an individual reports post-exertional malaise for 24 hours following an exercise program and their heart rate reached 140bpm/80% of their predicted HR max, they will be instructed to reduce their heart rate intensity to 110bpm/60% of their predicted HR max for their next exercise session).

Rehabilitation: The target behaviour to work towards participation in 150 minutes moderate intensity aerobic exercise per week as well as two resistance sessions, as per the above ACSM guidelines.

Telehealth supported: A physiotherapist will offer supervised weekly sessions with each participant via telehealth (or in person at RPH) to supervise exercises, adjust their programs as needed and to implement behaviour change strategies to aid uptake of optimal exercise habits throughout their BC treatment. Participants will also receive email or phone prompts to complete their program on days according to their exercise routine.

Those randomised to the control group will receive usual care. This comprises education regarding the importance of exercise during cancer treatment, a pamphlet and information regarding appropriate off-site exercise groups. In addition, the control group will receive a phone call from the research team once a month. During this phone call, information regarding healthcare utilisation will be collected and participants will receive generic encouragement to stay active during over the 16-week period. The phone call in the control group is important to reduce the potential confounding effect of increased contact (attention) with healthcare professionals in the experimental group.

3 Trial Aims / Objectives / Hypotheses

For all research questions, the study population refers to adults with newly diagnosed with breast cancer who are undergoing treatment through the Breast Clinic at RPH.

Primary research questions:

1) Does the SMART program, compared with usual care, change health-related quality of life (primary outcome) measured 16-weeks and 12-months following randomisation?

2) Does the SMART program, compared with usual care, change peripheral muscle strength, muscle mass, exercise tolerance, exercise adherence and chemotherapy completion rates (secondary outcomes) measured 16 weeks following randomisation?

Secondary research questions:

1) Do factors such as adherence, treatment pathway and cancer stage moderate the magnitude of any change in primary or secondary outcomes? (i.e. moderator analysis)

2) What is the health care utilisation, absenteeism and presenteeism over the 12 months following randomisation? (descriptive analyses only, to guide patient expectations in regarding these outcomes)

3) Does performance on tests of peripheral muscle force-generating capacity and exercise tolerance, change with test repetition (i.e. is there a learning effect)? (can be answered by Honours students enrolled in the BSc physiotherapy at Curtin University)

4) What is the minimal detectable difference in health-related quality of life and exercise tolerance? (can be answered by Honours students enrolled in the BSc physiotherapy at Curtin University)

5) Do psychological determinants of behavioural change, such as capability, opportunity, and motivation affect adherence to the SMART program and physical activity maintenance?

6) Does the SMART program improve mood, and does mood influence adherence to the program and physical activity maintenance?

4 Trial Design

Study Endpoints

The primary endpoint is health-related quality of life measured 16-weeks following randomisation (i.e. end of the intervention period).

Secondary endpoint is health-related quality of life measured 12-months following randomisation.

Study Design

A randomised controlled clinical trial will be conducted. Figure 1 illustrates the participant flow in the chart below.

Figure 1: Participant flow chart in the SMART exercise program study

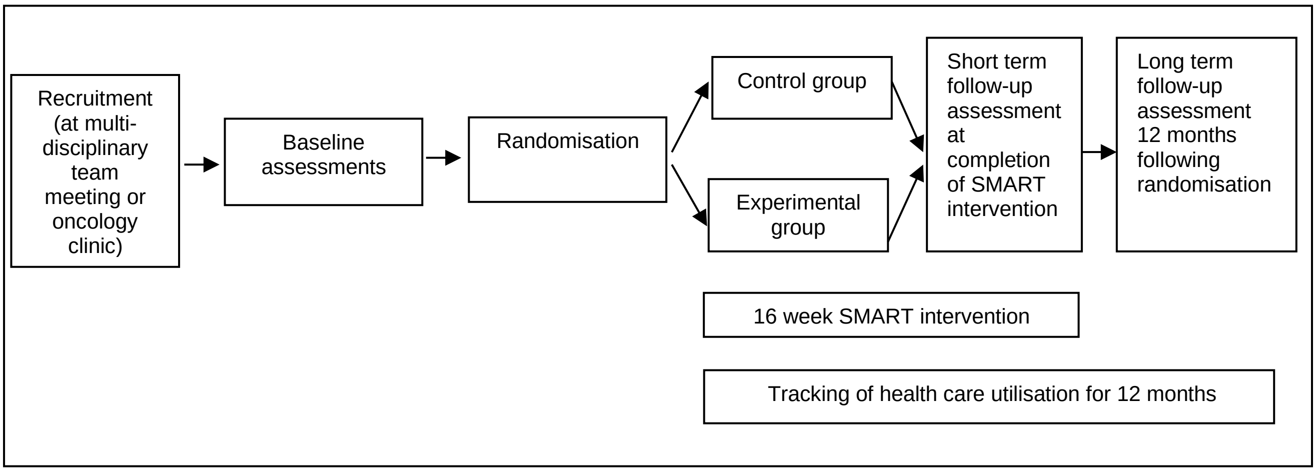

Bias

To reduce the bias, three strategies will be implemented. First, the personnel responsible for recruitment will be blinded to the randomisation (i.e. the randomisation sequence will be concealed). Second, to minimise contamination, separate groups of clinicians will carry out the SMART program and the usual care. Finally, assessors blinded to the group allocation will conduct all assessments following randomisation.

Blinding and Randomisation

Participants will be randomised (ratio of 1:1) to the experimental or control group, with computer generated randomisation using REDcap software. The randomisation sequence will be stratified for, (i) treatment pathway (chemotherapy, hormonal treatment and/or surgery), (ii) whether the person lives in Perth or outside Perth (i.e. rural or remote location) and (iii) whether the person regularly engaged in

exercise (or not) prior to their BC diagnosis. The randomisation sequence will be concealed using the REDcap software.

Data collected during all re-assessments (i.e. following randomisation) will be performed by a researcher blinded to group allocation.

Intervention/Product Description

The active ingredients of SMART program are described below. These will be delivered in an adaptable way; specific to each individual, periodised according to any treatment symptoms and progressed/regressed accordingly.

1) Exercise prescription

The target behaviour of the SMART program is for participants to work towards or maintain 150 minutes per week of moderate intensity aerobic exercise (or 75 minutes per week of vigorous intensity aerobic exercise), as well as two resistance training sessions per week.

Moderate intensity aerobic exercise will be defined as exercise performed at 60 to 75% of their age predicted maximum heart rate. Vigorous intensity aerobic exercise will be defined exercise performed at > 75% of their age predicted maximum heart rate.

Resistance training session will encompass the major muscle groups in 30 minutes of training and will be defined as completion of 3 sets of 8-12 repetitions of strength-based or functional tasks (for example, sit to stand, bicep curls, squats). The goal will be to report an intensity that is perceived a 15 (‘hard’) on the 6 to 20 Rating of Perceived Exertion. Intensity will be progressed by increasing the resistance and/or to a more challenging movement pattern.

2) Weekly sessions with a physiotherapist

Once a week, participants will meet with a physiotherapist, either in person (at RPH) or via telehealth. This session will be approximately one-hour in duration. During this session, (i) the participant and physiotherapist will reflect on the achievement of the goals set towards the target behaviour for the previous week, (ii) the participant will perform a supervised exercise session that comprises aerobic and resistance exercise and the physiotherapist will provide feedback and reassurance and, (iii) the participant and physiotherapist will plan the exercise training sessions for the following week.

Goal setting will be individualised and consider; (i) participant’s preferences and, (ii) the timing and severity of the likely or experienced side effects of the BC treatment. Goals will be agreed upon that are Specific, Measurable, Achievable, Relevant and Time-bound (SMART). An example of an exercise plan is included in Table 1 below.

Table 1: Example exercise plan

My exercise plan for week beginning 03/06/24:		
Monday	2pm	Gym session 60 mins (20-30 mins aerobic, 30 mins resistance exercises). Refer to your online exercise prescription.
Tuesday	10am	Walk dog for 45 minutes (aim for heart rate of 120bpm)
Wednesday		Walk dog for 45 minutes (aim for heart rate of 120bpm)<br>Chemo in the afternoon
Thursday	10am	Gym session 60 mins (20-30 mins aerobic, 30 mins resistance exercises). Refer to your online exercise prescription.
Friday		Day of rest

Saturday		Day of rest
Sunday		Day of rest, or consider one of your low intensity programs (30 min light walk aiming for heart rate of 90bpm or your online yoga program), if you are starting to feel better after your chemo this week.
**Don't forget to log your exercises on your online diary*		

3) Monitoring

Participants will be asked to monitor their adherence with exercise using an exercise diary. They will also be given a heart rate monitors to use while exercising so that they are able to track their exercise intensity and document these in the diary. An online platform (REDCap) will send prompts to participants to complete questionnaires relating to the side effects of their BC treatment and/or side effects from exercise sessions to aid in modulation of their treatment program.

4) Prompts and nudges

The participant will be able to opt-in to either online or phone messages reminding them of the exercise plan on any given day with scheduled exercise. Participants will also be encouraged to consider 'restructuring their environment' to provide prompts or reminders to exercise (e.g. place hand weights near the television, place their exercise shoes by the front door, set alarms on their phone to remind them to go for a walk etc).

5) Optimisation of social support

Following randomisation, participants will be invited to nominate a key support person (family member, friend, other) for the period of the trial. The support person will be invited to attend the baseline assessment and one-on-one sessions. Information regarding the importance of exercise across cancer treatment and strategies to facilitate this will be shared with the key support person. The role of the support person will be discussed and agreed upon by participant and support person, guided by the treating clinician.

6) Access to additional online support

An online digital resource (Physitrack) will contain individual exercise program(s) individual behaviour change strategies to aid in the uptake of the exercise. This online resource is currently used as a standard exercise prescription tool in the physiotherapy department at RPH. In addition, it will contain links to resources and advice relevant to individuals with breast cancer including psychological/emotional support resources, diet and nutrition education and education regarding referral pathways into available allied health services at RPH. As Physitrack requires participant mobile and/or email details for log-in purposes and to send exercise programs, there will be a section in the participant consent form with a check box allowing participants to opt in to this resource.

7) Optimisation of behaviour change technique use

An online digital workshop will contain advice for participants on identifying and effectively using behaviour change techniques based on the Behaviour Change Taxonomy (Michie et al., 2013); for example, action planning, problem solving, and habit formation. Additional booster sessions (over phone or telehealth) will also be available to participants, if appropriate.

Product Accountability Procedures

Physitrack is currently used by the RPH Physiotherapy department as a part of routine exercise prescription. This will be made available to all participants in the SMART program to aid exercise prescription and adherence tracking. REDCap is also currently used by RPH physiotherapy department.

Trial Duration/Schedule

The trial will run over a three-year period, with recruitment commencing in early 2024. Each participant will provide informed consent, after which they will complete a baseline assessment. On completion of the baseline assessments, participants will be randomised to either the experimental or control group and complete a 16-week intervention phase. Thereafter, all participants will complete a suite of re-assessments, (i) mid-way through the intervention phase, (ii) on completion of the intervention phase and, (iii) 12 months following randomisation.

Milestones	Timeline
1. Recruit study personnel	July 2023
2. Complete draft study proposal	November 2023
3. Submit to ethics/governance	November 2023
4. Approval obtained from all relevant HREC / RGO	February 2024
5. Commence recruitment (recruitment target = 15 participants/month)	March 2024
6. Commence intervention	March 2024
7. Last participant recruitment	March 2026
8. Complete intervention	July 2026
9. Complete post-intervention assessments	August 2026
10. Complete long term follow-up assessments	March 2027
11. Data cleaning, analysis and report writing	From March 2026
12. Final study publications and report complete	September 2027

Trial Termination

We will establish a Data Safety Monitoring Board (DSMB) of experienced researchers (n = 3), who are independent to the study group. This DSMB will meet after the successful recruit of each block of 50 trial participants. Progress of the trial participants (and any adverse events) and the changes in the primary outcome will be discussed. The DSMB will inform the study team of any concerns that would warrant discontinuation of the trial.

Data identification

Following informed consent, participants will be allocated a study number. This number will be used in lieu of any identifying information on any data exported from REDCap.

5 Source and Selection of Participants

Source of Participants

Participants will be recruited from the outpatient clinics at RPH. Those who appear to meet the study criteria will be identified in the weekly multi-disciplinary Breast Clinic team meetings. A nurse practitioner and/or other BC staff will approach those who meet criteria to share information regarding the trial.

There are some patients who complete radiation treatment prior to being given their script for their hormone medication by the radiation oncologist. The radiation treatment is completed outside RPH as there are no radiation oncology services onsite. For those patients receiving radiation oncology at SCGH, first contact may be made by the treating radiation oncologist to share information regarding the trial. It is noted that although first contact may occur at SCGH, these potential participants sit under the RPH Breast Clinic umbrella, i.e. those who were identified in the weekly RPH multi-disciplinary breast clinic team meetings following breast surgery at RPH but require adjuvant radiation treatment.

Those who express an interest will be contacted by a member of the research team, who will discuss the study with the potential participant. Informed consent will be gained prior to participation in the study.

Participant inclusion criteria

Adults will be eligible to participate if they meet the following criteria:

1. Adult with newly diagnosed breast cancer (within the last 6 months)
2. Consent to treatment for their BC, which may include chemotherapy and/or endocrine therapy

Participant exclusion criteria

Adults will be not eligible to participate if they meet any of the following criteria:

1. Cognitive impairment which limits ability to complete assessments and/or adhere to an independent exercise regime;
2. Unable to access internet/phone application;
3. Living in supported residential care;
4. Any co-morbid condition where exercise is contra-indicated (as determined by the treating specialist).

Participant withdrawal criteria

(i.e. terminating investigational product/trial treatment) and procedures specifying:

(a) when and how to withdraw participants from the project;
Participants can ask to withdraw from this study at any time. To do so, they need to inform a member of the research team that no longer want to be involved. Contact details of the research team will be provided in the Participant Information Form. Withdrawal from the study would not mean a withdrawal of treatment. i.e., participants can still access usual care and no impact on any future BC treatment.

(b) the type and timing of the data to be collected for withdrawn participant(s);
We will ask any participant who chooses to withdraw from the study if we can retain any data already collected.

(c) whether and how participants are to be replaced; and
We will continue to recruit until the required sample size has completed the 16-week intervention period.

(d) the follow-up for participants withdrawn from the project.
Nil – other than usual care.

6 Treatment of Participants

Description and justification for treatments, interventions or methods to be utilised

Those randomised to the experimental group will receive the *SMART* exercise program, as discussed above. Those randomised to the control group will receive usual care. This comprises education regarding the importance of exercise during cancer treatment, a pamphlet/information regarding appropriate off-site exercise groups. Separate oncology physiotherapy provision for patients undergoing chemotherapy treatment involves education strategies and the option to attend a weekly exercise session in the physiotherapy gym. However, exercise intervention as part of routine physiotherapy care for this patient group at RPH is currently through an “opt-in” system and resultantly uptake of this service is low. In addition, the control group will receive a phone call from the research team once a month. During this phone call, information regarding healthcare utilisation will be collected and participants will receive generic encouragement to stay active during over the 16-week period.

The trial will run over a three-year period, with recruitment commencing in early 2024. Each participant will provide informed consent, after which they will complete a baseline assessment. On completion of these baseline assessments, participants will be randomised to either the experimental or control group and complete a 16-week intervention phase. Thereafter, all participants will complete a suite of re-assessments, (i) mid-way through the intervention phase, (ii) on completion of the intervention phase and, (iii) 12 months following randomisation.

Permitted medications/treatments

This trial will not impact medication use. There are no inclusion/exclusion criteria based on medications.

7 Assessment of Efficacy

After recruitment, all study participants will complete an initial assessment to establish their baseline measures. The participants will be requested to complete the EORTC C30 questionnaire, as well as the breast cancer specific BR45 module, the EQ-5D-5L quality of life questionnaires and questionnaires measuring psychological determinants of behaviour change. The trial physiotherapist will also assess participant muscle strength (grip strength, upper limb and lower limb muscle strength) and exercise tolerance (6-minute walking distance). Participant body composition will also be measured using the SOZO analyser. These outcomes are discussed in detail below. This assessment will be repeated at the end of the intervention.

PRIMARY OUTCOMES

Health-related quality of life will be measured using the EQ-5D-5L questionnaire and the EORTC Quality of Life C30 questionnaire together with the breast cancer specific BR45 module. These questionnaires have been validated for use in the BC population (Montagnese et al, 2021).

SECONDARY OUTCOMES

Peripheral muscle force-generating capacity will be measured as; (i) grip strength, (ii) force generated during a maximum voluntary isometric contraction of the middle deltoids, biceps brachii and quadriceps femoris. These are described below.

> *Grip strength* will be measured using a Jamar hand-held dynamometer (Surgical Synergies, SI Instruments, SA, Australia). The participant will perform the test in sitting. Starting position will be shoulder in adduction, elbow flexion to 90 degrees, forearm and wrist in neutral position. The participant will be encouraged to perform a maximal squeeze of the dynamometer for ~3 seconds. The test will be repeated 5 times (on each hand) and the measure that is the highest, but within 10% of one other will be recorded as the test result (Gittings, et al, 2018).
>
> *Middle deltoids and biceps brachii strength* will be assessed using a Lafayette Muscle Meter no. 01165 (SI Instruments, SA, Australia). The participant will perform the test in sitting. Starting

position will be shoulder in adduction and neutral external rotation, elbow flexion to 90 degrees, forearm and wrist in neutral position. The participant will be encouraged to abduct their shoulder (for middle deltoids) or flex their elbow (for biceps brachii) with as much force as possible against the dynamometer for 3 seconds. The test will be repeated 5 times (on each arm) and the measure that is the highest, but within 10% of one other will be recorded as the test result (Gittings, et al, 2018).

Quadriceps femoris strength will be measured using a Lafayette Muscle Meter no. 01165 (SI Instruments, SA, Australia). The participant will perform the test in sitting. Starting position will be hip and knee flexion to 90 degrees. The participant will be encouraged to extend their knee with as much force as possible against the dynamometer for 3 seconds. The muscle meter will be stabilised with by the assessing therapist with the use of a nylon belt secured to the back leg of the chair, in order to maximise reliability. The test will be repeated 5 times (on each leg) and the measure that is the highest, but within 10% of one other will be recorded as the test result (Gittings, et al, 2018).

Exercise tolerance will be measured as the six-minute walk test (Schmidt 2013). This test will be performed according to standard guidelines (Schmidt 2013). The outcome measured is the distance walked in 6 minutes (6MWD). Each participant will perform the test twice and the longest 6MWD will be recorded as the test result.

Muscle mass and body composition will be measured via bioimpedance spectroscopy. This is a non-invasive measure of body composition performed using the SOZO analyser (Impedimed, Brisbane, Queensland). The test takes under a minute, with the participant clothed and standing on the testing platform. The SOZO devide is already used in standard care in patient with BC to identify the presence of lymphoedema. This device applies 256 discrete current frequencies through the body, which provides estimates of tissue composition (skeletal muscle mass, fat mass, fat free mass), fluid variables (total body water, extracellular fluid and intracellular fluid), and a metabolic report (cell health, basal metabolic rate and active tissue mass) (Kyle, et al, 2004; Grisbrook, et al, 2015).

Chemotherapy completion rates will be extracted from medical records and recorded as a mean relative dose intensity (RDI), which records the actual chemotherapy medication dose intensity as a fraction of the original planned dose intensity, accounting for both the dose and the number of weeks in the chemotherapy cycle (Longo 1991). Any dose adjustments or dose delays will be obtained from the medical records and/or the treating oncologist and the percent of participants requiring dose adjustments or delay will be recorded.

Psychological determinants of behaviour change will be measured using; (i) the Capability, Opportunity, Motivation – Behaviour (COM-B; Michie et al. 2011) model factors, (ii) impairment in functioning, (iii) intention, (iv) self-efficacy, (v) habit. These are described below:

COM-B framework factors will be measured using the COM questionnaire (Keyworth et al., 2020). The COM questionnaire is a questionnaire measuring people's perceptions of each component of the COM-B model on their performance of target behaviours (Michie et al. 2011): physical and psychological capability; physical and social opportunity; and reflective and automatic motivation. The questionnaire was designed to be adaptable to different behaviours and populations, including exercise engagement (Keyworth et al., 2020). The measure has been adapted for the current study to assess participants' perceived ability to achieve their exercise goals; for example, "*I have the **PHYSICAL** opportunity to achieve my weekly exercise goals (e.g., time, the necessary equipment, space)"*.

Impairment in functioning will be measured using the Work and Social Adjustment Scale (WSAS; Mundt et al., 2002). The WSAS is a questionnaire measuring the impact of someone's mental health on their ability to perform day-to-day functions, such as managing their home or engaging in leisure activities. The WSAS has demonstrated good validity in previous uses in the BC population (Lyons et al., 2015; Thandi et al., 2017).

Intention to exercise will be measured using two questions based on the theory of planned behaviour (Ajzen, 1991). The items will measure intention to perform aerobic exercise and resistance exercise with examples of each. Previous studies suggest that a single item per behaviour is sufficient to measure intention due to high correlations between items in lengthier measures, with the added benefit of reducing participant burden (Charlesworth et al., 2021; McAlpine & Mullan, 2022).

Self-efficacy will be measured using the Brief Self-Control Scale (BSCS; Tangney et al., 2004) and the Barrier and Task Self-Efficacy Scale (BTSES; Rogers et al., 2006). The BSCS is a validated questionnaire measuring general self-control that has often been used in studies exploring behaviour change (Allom et al., 2018). The BTSES is a questionnaire measuring self-efficacy around physical exercise developed to be used with the BC population and has shown good validity (Rogers et al., 2006).

Habit will be measured using the Habitual Tendencies Questionnaire (HTQ; Ramakrishnan et al., 2022) and the Self-Report Behavioural Automaticity Index (SRBAI; Gardner et al., 2012). The HTQ measures people's tendencies to rely on habits and has shown good validity when compared to similar measures such as the Routine subscale of the Creature of Habit Scale (Ersche et al., 2017; Ramakrishnan et al., 2022).The SRBAI measures people's perceptions of how automatic they find a specific behaviour, and has shown good validity when adapted to assess habitual physical exercise engagement (Phillips & Gardner, 2016; Weyland et al., 2022).

Mood will be measured using the Positive and Negative Affect Scale (PANAS; Watson 1988) questionnaire. The PANAS is a 20-item questionnaire of positive and negative mood that has been commonly used in the BC population, demonstrating high validity (Moreno et al., 2016; Voogt et al., 2005).

OTHER VARIABLES

Descriptive variables will be recorded such as age, sex, body mass index, employment status, co-morbid conditions, stage of cancer and details of cancer treatment.

Healthcare utilisation and adherence with exercise will be assessed in both groups. In the control group, this will be documented during the monthly phone calls and exercise diaries. In the experimental group, this will be monitored by asking each participant during the weekly supervised sessions and also using exercise diaries (see Section 4, Monitoring). Regarding healthcare utilisation, we will ask each participant to recall the number of; (i) visits to the Emergency Department, (ii) inpatient admission days, (ii) outpatient appointments and, (iii) visits to any other healthcare provider (Y/N, who, number of visits). Regarding adherence with exercise, we will ask each participant to recall the number of minutes spent engaging in moderate or vigorous intensity exercise and details regarding any resistance exercise.

Absenteeism and presenteeism will be assessed using the iMTA productivity cost questionnaire (Bouwmans 2015)**.**

Global rating of change will be assessed by asking participants, on completion of the SMART exercise program, to indicate if their health-related quality of life or exercise tolerance had 'improved', 'worsened' or 'stayed the same'. Those participants who rated their walking ability as 'improved' or 'worsened' will then be asked to rate the degree of change on a seven-point scale (1 a little better to 7 a great deal better).

Feasibility and acceptability will be measured using; (i) Quantitative data from an acceptability and feasibility questionnaire, and (ii) qualitative data from exit interviews. These are described below:

Quantitative data will be collected using a questionnaire asking participants to rate their perceptions of the SMART exercise program (e.g., "*I thought the SMART exercise program was easy to understand*"). Participants can rate each item on a five-point scale (1 "*I don't agree at all*" to 5 "*I totally agree*". Four open-ended items at the end of this questionnaire allow participants to include additional feedback (e.g., "*What did you **like** about the SMART exercise program?*").

Qualitative data will be collected using exit interviews upon completion of the SMART exercise program. Participants will have the opportunity to opt-in to semi-structured interviews to share more in-depth information about their experience with the SMART exercise program. Interviews will be conducted online and recorded using Teams, and are expected to last 30-45 minutes. Informed consent will be obtained from participants prior to the meeting and again before the interview or recording begins.

Efficacy assessment

Data collection will require two visits to the Physiotherapy Department at RPH. These assessments will be approximately 90 minutes in duration and will be completed pre-randomisation (baseline assessment) and at the end of the intervention phase. All other data collection will be done by extracting information from medical records, during phone conversations with the participants or by emailing out questionnaires through online formats (e.g. REDCap).

Table 1: Timing of outcomes

	**Pre-randomisation**	**Each week during the intervention phase**	**Mid-way through the intervention phase**	**On completion of the of intervention phase**	**12 months following randomisation**
Descriptive data (age, sex etc)	X				
EORTC QLQ C30 with BR45 (health-related quality of life)	X		X	X	X
EQ-5D-5L	X		X	X	X
Grip strength	X			X	
Deltoid, bicep and quads strength	X			X	
6MWT	X			X	
Muscle mass	X			X	
Chemotherapy completion rates		X		X	
COM	x		x	x	x
WSAS	x		x	x	x
Intention	x		x	x	x
BTSES	x		x	x	x
HTQ	x		x	x	x
SRBAI	x		x	x	x
PANAS	x		x	x	x
Participation in exercise		X			
Healthcare utilisation		X			
iMTA (absenteeism / presenteeism)			X	X	X
Acceptability/ feasibility				x	
Exit interview				x	

8 Assessment of Safety

Risks and benefits

This is a low risk study. Exercise during cancer treatments has been widely studied, reported as a safe intervention for individuals with cancer and is recommended as part of usual clinical care (Cormie 2018). Participants may experience some post exertional malaise and/or delayed onset muscle soreness, but the research team, trained physiotherapists, will ask about these symptoms every week and modify the program as required and also liaise with the treating medical team. The main risk to the participants in this study is inconvenience relating to the time and travel expense associated with attending the study assessments.

We will be assessing quality of life surveys. It is possible that participants will report low or reducing quality of life and/or depressive symptoms. Should these be identified we will recommend and refer the participant to the psychology support services, through the usual referral pathways within the Breast Clinic.

Regarding benefits, those allocated to the experimental group will receive the SMART program. We hope that this program will optimise participation in structured exercise and produce sustained improvements in health-related quality of life. If this is the case, we will endeavour to implement a similar program as part of routine physiotherapy care at RPH in the future.

Participants will be offered vouchers in compensation for travel costs for the face to face appointments and the time taken to complete the study assessments. These vouchers will be valued at $15 for travel cost compensation and $20 for time compensation to complete the assessments.

Safety

Physiotherapists are trained in providing exercise prescription for a wide variety of health conditions. All RPH physiotherapy staff have annual training to stay up-to-date with departmental emergency procedure training specific to gym based exercise sessions and telehealth appointments run through the RPH. Nevertheless, during each weekly contact, for participants in both groups, we will record (in REDCap) for any adverse events and classify these as minor (i.e. resolve without any need for a medical review) or non-minor (i.e. those that warranted a medical review).

Examples of minor adverse events might include episodes of dizziness or nausea during exercise or the muscle soreness and generalise malaise on completion of an exercise session. Example of non-minor adverse events might include an injurious fall or cardiac event. These will be discussed at the DSMB.

Data and Safety Monitoring Board

A data and safety monitoring board (DSMB) will be set up for this research project to review trial data at regular intervals. The members of the DSMB will be independent to the clinical researchers actively working on the project. The planned DSMB members include a statistician from Curtin University, an academic working (with experience in BC research) from an interstate university and a BC consumer advocate.

Adverse event reporting

Non-minor adverse events will be reported to the HREC within 48 hours.

Follow-up of Adverse Events

We will report any adverse events to the treating medical team as appropriate.

9 Data Management, Statistical Analysis and Record Keeping

Statistics and Interim Analysis

The primary research questions for this study are:

- Does the SMART program, compared with usual care, change health-related quality of life (primary outcome) measured 16-weeks and 12-months following randomisation?

AND

- Does the SMART program, compared with usual care, change peripheral muscle strength, muscle mass, exercise tolerance, exercise adherence and chemotherapy completion rates (secondary outcomes) measured 16 weeks following randomisation?

The analyses for this research question will be performed according to the intention to treat principle. For the primary outcome, health-related quality of life, with 4 assessment points, linear models will be used to account for repeated measures with participant ID entered as a random effect. The primary time point of interest will be the end of the intervention phase. Group, time and group by time will be analysed as fixed effects and the baseline measure will be entered as a covariate to adjust for random baseline differences. Between group difference for the dependent outcome of chemotherapy completion (yes/no) will be analysed using logistic regression. Between group differences for the remaining secondary measures (peripheral muscle force-generating capacity, muscle mass, exercise tolerance) will be examined using linear regression with the corresponding baseline measure used as a covariate. Data transformations or alternative models (such as gamma distribution) will be considered if there is poor model fit.

Missing data will be examined and multiple imputations investigated (assuming they are missing at random). Models will be adjusted for covariates, randomisation variables and confounders such as age. Estimates of the effects will be reported with their corresponding 95% confidence intervals.

The first secondary research question is:

- Do factors such as adherence, treatment pathway and cancer stage moderate the magnitude of any change in primary or secondary outcomes? (i.e. moderator analysis)

To understand any moderator effects, the listed covariates will be entered into a multivariable model using the outcomes listed above. In addition, adherence will be examined to see if it mediates the relationship with the outcomes, by calculating the indirect through the use of partial coefficients.

The second secondary research question is:

- What is the health care utilisation, absenteeism and presenteeism over the 12 months following randomisation?

The is an exploratory question and data will be described using mean and standard deviation.

The third secondary research question is:

- Does performance on tests of peripheral muscle force-generating capacity and exercise tolerance, change with test repetition (i.e. is there a learning effect)?

This will be assessed using one-way repeated measures analysis of variance (ANOVA). That is, for each participant, data collected over consecutive trials (5 attempts for each measure of muscle force and 2 attempts for the 6MWT) compared to explore if there is an effect of test repetition on these outcomes.

The fourth secondary research question is:

What is the minimal detectable difference in health-related quality of life and exercise tolerance?

This will be assessed using anchor-based approach via a Receiver Operating Characteristic curve. This curve will determine the optimal operating point to discriminate between participants who rate themselves as changed vs unchanged at the end of the SMART program.

The fifth secondary research question is:

Do psychological determinants of behavioural change, such as capability, opportunity, and motivation affect adherence to the SMART program and physical activity maintenance?

This will be assessed using correlations and a Multiple Regression Analysis of the data from psychological determinant measures (COM-B factors, impairment in functioning, intention, self-efficacy, habit, and mood) across all time-points for each participant. Data from the exit interviews will also be analysed using qualitative methods (such as thematic analysis) to explore participants' reasons for engagement (or lack of engagement).

The sixth secondary research question is:

Does the SMART program improve mood, and does mood influence adherence to the program and physical activity maintenance?

This will be assessed using correlations between mood data from the PANAS (across all timepoints) and physical activity engagement.

Sample Size

Sample size calculation has been conducted using the EORTC QLQ BR-30 (continuous data) as the dependent variable. To detect a difference between the experimental and control groups in this outcome of 8 (which has been estimated as the minimal clinically important difference), assuming a standard deviation of 20.52 (based on previously published literature) (Karsten 2022), with an $\alpha = 0.05$ and $1-\beta = 0.8$, we will need to 104 participants in the experimental group and 104 participants in the control group. In order to account for 20% drop-out, we plan to recruit a sample size of 130 participants in each group. This means we aim to recruit 260 participants over 24 months (recruitment rate of 11 per month).

We believe that this recruitment rate is realistic. This is because, we have recently completed an audit of the RPH Breast Clinic (GEKO number 50167). The Data, Digital and Innovation (DDI) group at RPH compiled information from WEBPAS and showed that 993 adults were treated with surgical breast procedures and/or chemotherapy treatment for BC at the RPH Breast Clinic between 01/07/2021 to 30/06/2023. This is equivalent to 41 people per month. This means that our recruitment fraction for this study will be 27%.

Study Power and Significance

$\alpha = 0.05$ and $1-\beta = 0.8$

Statistical plan deviations:

The final statistical analyses plan (SAP) will be published when we publish the study protocol. Any deviations from the SAP will be described in the final manuscript.

Selection of participants for analyses:

All randomised participants will be included in the final analyses, according to the intention to treat principle.

Data management

Collection: Data collection will be undertaken using the REDCap software.

Storage: Data is captured using the REDCap software (licenced to Curtin University).. Only coded data, with all identifiers removed (e.g. name, date of birth) will be entered into REDCap. Data, without any identifiers, will be exported and stored on the W drive at RPH. The Master Log which links the

participant’s identity to their study code will be stored separately in a password protected folder on the secure WA health server. Access to this Master Log will be restricted to the study researchers who are WA Health employees.

In addition, a program specific to this study will be used to export and store information from the wearable monitors data relating to heart rate and activity metrics. This program will be specific to this study will be set-up through the HIVE department at RPH, using existing software and data (without any identifiers) will stored within the RPH server.

Maintenance: The servers at RPH and Curtin University are backed up regularly.

Security: There are several security features both within the REDCap software itself, and also the network and server hosting this software. Only REDCap users (investigators) associated with this project will be granted user privileges within the software. REDCap maintains and build an audit trail so all users will have all activity tracked. . Similarly, only investigators associated with this project will be granter user privileges to the program storing data from the wearable devices. Log on to REDCap software and the program relating to the wearable data sit behind RPH firewall and requires all users to verify their identity using authenticator codes. REDCap contains an auto-logout which will log a user out after 30 minutes of inactivity

Archiving: Data will be stored for 7 years. Following this time, electronic files will be deleted.

Procedures for missing, unused and spurious data:

Data will be collected in REDCap and checked weekly for missing or spurious data. Further, REDCap has the capacity to limit spurious values for specific questions (e.g. height [m] must have values than range between 1.3 and 2.5 so you cannot enter height in cm).

11 Monitoring / Audit

Monitoring, Audit and Regulatory Inspections Statement

This project has an established steering committee, which comprises representatives from RPH, Curtin University and consumer representatives. We meet monthly via teleconference (and will continue to so over the life of the trial) to discuss conduct of this study.

The CPI will review data regularly (weekly) through REDCap.

Procedures for monitoring and auditing

In addition to the processes described above, we will also provide data to the DSMB every 3 months for review. This will provide another time point to ensure all data have been entered into REDCap correctly.

12 Quality Control and Quality Assurance

Compliance statement.

All aspects of this project will be undertaken in accordance with this protocol and follow Good Clinical Practice recommendations.

13 Ethics

Ethics approval will be sought from the RPH Human Research Ethics Committee.

All participants will be required to provide written informed consent prior to participation. These signed consent forms will be stored in the Physiotherapy Department (in a locked office) at RPH.

14 Budget, Financing, Indemnity and Insurance

Personnel involved in data collection will be employed through RPH. As such, RPH will provide insurances such as public liability and professional indemnity. The budget for this study has been funded by the *Vonesch Breast Cancer Rehabilitation Grant Agreement*. A separate budget document has been included as supplementary information to this form.

15 Publication

This study has been submitted for registration with the Australian New Zealand Clinical Trial Registry (ANZCTR), registration number ACTRN12623001168640p.

A framework for knowledge translation will be drafted at the commencement of the project and continually adapted as the project progresses (e.g. in response to consumer-input and new knowledge). Preparatory work towards this initial draft of this framework has included consideration of the following factors:

1) ***What are likely to be the most important findings?***
   - The effect of the SMART program on a range of patient centred outcome measures.
   - The minimal detectable difference for measures of health-related quality of life and exercise tolerance in this patient population.
   - Information regarding optimisation of measures of muscle force and exercise tolerance.
   - Descriptive data on absenteeism and presenteeism.
   - The effects of individual differences in psychological determinants of behaviour change (COM-B factors, impairment in functioning, intention, self-efficacy, habit, and mood) on physical adherence and maintenance.

2) ***Who needs to be aware of this information?***
   - Healthcare professionals (e.g. physicians, general practitioners, nurses, allied health) who interact with people with BC
   - People with BC and people who support / care for those with BC.
   -

3) ***What strategies will be used to engage the appropriate people in the process of knowledge translation?***

   Healthcare professionals and researchers: Strategies will include; (i) publication in peer-reviewed journals, (ii) communication with professional bodies the Breast Cancer Network Australia and the Australian Physiotherapy Association, (iii) professional development and staff education events at RPH as well as Curtin University.

4) ***What are the possible key conferences / professional events that need to be targeted?***

**Conference/event**	**Target audience**
Breast Cancer Trials annual scientific meeting	HCP/researchers with an interest in BC (in Australia)
Australian Physiotherapy Association	Physiotherapists (in Australia)
COSA Annual Scientific Meeting	HCP/researchers with an interest in BC (in Australia)
Living well with breast cancer online information forums run by the Breast Cancer Network Australia	People with BC
Australasian Society for Behavioural Health and Medicine (ASBHM) annual scientific conference	Researchers with an interest in behaviour change (Australia)

European Health Psychology Society (EHPS) annual conference	Researchers with an interest in behaviour change
Western Australia Psychological Science Conference (WAPSC)	Researchers with an interest in behaviour change (Australia)

16 References

AJZEN, I. THE THEORY OF PLANNED BEHAVIOR. ORGANIZATIONAL BEHAVIOR AND HUMAN DECISION PROCESSES. 1991;50(2):179-211.

ALLOM, V., MULLAN, B. A., MONDS, L., ET AL. REFLECTIVE AND IMPULSIVE PROCESSES UNDERLYING SAVING BEHAVIOR AND THE ADDITIONAL ROLES OF SELF-CONTROL AND HABIT. JOURNAL OF NEUROSCIENCE, PSYCHOLOGY, AND ECONOMICS. 2018;11(3);135.

BOUWMANS C, KROL M, SEVERENS H, ET AL. THE IMTA PRODUCTIVITY COST QUESTIONNAIRE: A STANDARDIZED INSTRUMENT FOR MEASURING AND VALUING HEALTH-RELATED PRODUCTIVITY LOSSES. VALUE HEALTH. 2015;18(6):753-8.

CANCER AUSTRALIA. BREAST CANCER IN AUSTRALIA STATISTICS. ACCESSED ON 26/10/23. FROM: BREAST CANCER IN AUSTRALIA STATISTICS | CANCER AUSTRALIA

CHARLESWORTH, J., MULLAN, B., & MORAN, A. INVESTIGATING THE PREDICTORS OF SAFE FOOD HANDLING AMONG PARENTS OF YOUNG CHILDREN IN THE USA. FOOD CONTROL. 2021;126:108015.

CLINICAL ONCOLOGY SOCIETY OF AUSTRALIA. COSA POSITION STATEMENT ON EXERCISE IN CANCER CARE. APRIL 2018.

ERSCHE, K. D., LIM, T.-V., WARD, L. H. E., ET AL. CREATURE OF HABIT: A SELF-REPORT MEASURE OF HABITUAL ROUTINES AND AUTOMATIC TENDENCIES IN EVERYDAY LIFE. PERSONALITY AND INDIVIDUAL DIFFERENCES. 2017;116:73-85.

FERLAY J, ERVIK M, LAM F, ET AL. 2020. GLOBAL CANCER OBSERVATORY: CANCER TODAY. LYON, FRANCE: INTERNATIONAL AGENCY FOR RESEARCH ON CANCER.

FERREIRA AR., DI MEGLIO A., PISTILLI B., ET AL. DIFFERENTIAL IMPACT OF ENDOCRINE THERAPY AND CHEMOTHERAPY ON QUALITY OF LIFE OF BREAST CANCER SURVIVORS: A PROSPECTIVE PATIENT-REPORTED OUTCOMES ANALYSIS. ANN ONCOL. 2019:30, 1784-1795.

FERRI, A., GANE, E. M., SMITH, M. D., ET AL. EXPERIENCES OF PEOPLE WITH CANCER WHO HAVE PARTICIPATED IN A HOSPITAL-BASED EXERCISE PROGRAM: A QUALITATIVE STUDY. SUPPORTIVE CARE IN CANCER. 2021;29(3): 1575-1583.

FICARRA S, THOMAS E, BIANCO A, ET AL. IMPACT OF EXERCISE INTERVENTIONS ON PHYSICAL FITNESS IN BREAST CANCER PATIENTS AND SURVIVORS: A SYSTEMATIC REVIEW. BREAST CANCER. 2022;29(3):402-418.

FITZMAURICE C., ALLEN C, BARBER RM, ET AL. GLOBAL, REGIONAL, AND NATIONAL CANCER INCIDENCE, MORTALITY, YEARS OF LIFE LOST, YEARS LIVED WITH DISABILITY, AND DISABILITY-ADJUSTED LIFE-YEARS FOR 32 CANCER GROUPS, 1990 TO 2015: A SYSTEMATIC ANALYSIS FOR THE GLOBAL BURDEN OF DISEASE STUDY. JAMA ONCOL. 2017;3, 524-548.

FRANZOI MA., AGOSTINETTO E, PERACHINO M, ET AL. EVIDENCE-BASED APPROACHES FOR THE MANAGEMENT OF SIDE-EFFECTS OF ADJUVANT ENDOCRINE THERAPY IN PATIENTS WITH BREAST CANCER. LANCET ONCOL. 2021;22, E303-E313.

GARDNER, B., ABRAHAM, C., LALLY, P., ET AL. TOWARDS PARSIMONY IN HABIT MEASUREMENT: TESTING THE CONVERGENT AND PREDICTIVE VALIDITY OF AN AUTOMATICITY SUBSCALE OF THE SELF-REPORT HABIT INDEX. INTERNATIONAL JOURNAL OF BEHAVIORAL NUTRITION AND PHYSICAL ACTIVITY. 2012;9(1):1-12.

GIAQUINTO AN, SUNG H., MILLER KD, ET AL. BREAST CANCER STATISTICS, 2022. CA CANCER J CLIN. 2022;72, 524-541.

GILDEA GC, SPENCE RR, JONES TL, ET AL. BARRIERS, FACILITATORS, PERCEPTIONS AND PREFERENCES INFLUENCING PHYSICAL ACTIVITY PARTICIPATION, AND THE SIMILARITIES AND DIFFERENCES BETWEEN CANCER TYPES AND TREATMENT STAGES - A SYSTEMATIC RAPID REVIEW. PREV MED REP. 2023;24;34:102255.

GRISBROOK T, KENWORTHY P, PHILLIPS M., ET AL. ALTERNATE ELECTRODE PLACEMENT FOR WHOLE BODY AND SEGMENTAL BIOIMPEDANCE SPECTROSCOPY. PHYSIOL MEAS. 2015;36(10), 2189-2201.

GITTINGS PM, HINCE DA, WAND BM, ET AL. GRIP AND MUSCLE STRENGTH DYNAMOMETRY IN ACUTE BURN INJURY: EVALUATION OF AN UPDATED ASSESSMENT PROTOCOL. JOURNAL OF BURN CARE & RESEARCH. 2018;39(6), 939-947.

KARSTEN MM, ROEHLE R, ALBERS S, ET AL. REAL-WORLD REFERENCE SCORES FOR EORTC QLQ-C30 AND EORTC QLQ-BR23 IN EARLY BREAST CANCER PATIENTS. EUR

J CANCER. 2022 MAR;163:128-139. DOI: 10.1016/J.EJCA.2021.12.020. EPUB 2022 JAN 20. ERRATUM IN: EUR J CANCER. 2022;174:325-327.

KEYWORTH, C., EPTON, T., GOLDTHORPE, J., ET. ACCEPTABILITY, RELIABILITY, AND VALIDITY OF A BRIEF MEASURE OF CAPABILITIES, OPPORTUNITIES, AND MOTIVATIONS (“COM-B”). BRITISH JOURNAL OF HEALTH PSYCHOLOGY. 2020;25(3): 474-501.

KYLE UG, BOSAEUS I, DE LORENZO AD, ET AL. BIOELECTRICAL IMPEDANCE ANALYSIS—PART I: REVIEW OF PRINCIPLES AND METHODS. CLINICAL NUTRITION. 2004;23(5):1226-1243.

LONGO DL, DUFFEY PL, DEVITA VT JR ET AL. THE CALCULATION OF ACTUAL OR RECEIVED DOSE INTENSITY: A COMPARISON OF PUBLISHED METHODS. J CLIN ONCOL 1991;9:2042–2051.

LYONS, K. D., SVENSBORN, I. A., KORNBLITH, A. B., ET AL. A CONTENT ANALYSIS OF FUNCTIONAL RECOVERY STRATEGIES OF BREAST CANCER SURVIVORS. OTJR: OCCUPATION, PARTICIPATION AND HEALTH. 2015;35(2):73-80.

MCALPINE, T., & MULLAN, B. A. THE ROLE OF ENVIRONMENTAL CUES IN SUGAR-SWEETENED BEVERAGE CONSUMPTION USING A TEMPORAL SELF-REGULATION THEORY FRAMEWORK. APPETITE. 2022;169:105828.

MENESES-ECHÁVEZ JF, GONZÁLEZ-JIMÉNEZ E, RAMÍREZ-VÉLEZ R. EFFECTS OF SUPERVISED EXERCISE ON CANCER-RELATED FATIGUE IN BREAST CANCER SURVIVORS: A SYSTEMATIC REVIEW AND META-ANALYSIS. BMC CANCER. 2015;21:15:77.

MICHIE S, RICHARDSON M, JOHNSTON M, ET AL. THE BEHAVIOR CHANGE TECHNIQUE TAXONOMY (V1) OF 93 HIERARCHICALLY CLUSTERED TECHNIQUES: BUILDING AN INTERNATIONAL CONSENSUS FOR THE REPORTING OF BEHAVIOR CHANGE INTERVENTIONS. ANN BEHAV MED. 2013;46(1):81-95.

MICHIE, S., VAN STRALEN, M. M., & WEST, R. THE BEHAVIOUR CHANGE WHEEL: A NEW METHOD FOR CHARACTERISING AND DESIGNING BEHAVIOUR CHANGE INTERVENTIONS. IMPLEMENTATION SCIENCE. 2011;6(1):42.

MISHRA SI, SCHERER RW, GEIGLE PM, ET AL. EXERCISE INTERVENTIONS ON HEALTH-RELATED QUALITY OF LIFE FOR CANCER SURVIVORS. COCHRANE DATABASE SYST REV. 2012;15;2012(8):CD007566.

MONTAGNESE C, PORCIELLO G, VITALE S, ET AL. QUALITY OF LIFE IN WOMEN DIAGNOSED WITH BREAST CANCER AFTER A 12-MONTH TREATMENT OF LIFESTYLE MODIFICATIONS. NUTRIENTS. 2020 DEC 31;13(1):136. DOI: 10.3390/NU13010136. PMID: 33396551; PMCID: PMC7824271.

MORENO, P. I., MOSKOWITZ, A. L., GANZ, P. A., & BOWER, J. E. POSITIVE AFFECT AND INFLAMMATORY ACTIVITY IN BREAST CANCER SURVIVORS: EXAMINING THE ROLE OF AFFECTIVE AROUSAL. PSYCHOSOMATIC MEDICINE. 2016;78(5): 532-541.

MUNDT, J. C., MARKS, I. M., SHEAR, M. K., ET AL. THE WORK AND SOCIAL ADJUSTMENT SCALE: A SIMPLE MEASURE OF IMPAIRMENT IN FUNCTIONING. BRITISH JOURNAL OF PSYCHIATRY. 2002;180(5):461-464.

PHILLIPS, L. A., & GARDNER, B. HABITUAL EXERCISE INSTIGATION (VS. EXECUTION) PREDICTS HEALTHY ADULTS’ EXERCISE FREQUENCY. HEALTH PSYCHOLOGY. 2016;35(1):69.

PLINSINGA ML, SINGH B, ROSE GL, ET AL. THE EFFECT OF EXERCISE ON PAIN IN PEOPLE WITH CANCER: A SYSTEMATIC REVIEW WITH META-ANALYSIS. SPORTS MED. 2023 SEP;53(9):1737-1752.

PORTENOY RK, THALER HT, KORNBLITH AB, ET AL. THE MEMORIAL SYMPTOM ASSESSMENT SCALE: AN INSTRUMENT FOR THE EVALUATION OF SYMPTOM PREVALENCE, CHARACTERISTICS AND DISTRESS. EUR J CANCER. 1994;30A(9):1326-36.

RAMAKRISHNAN, S., ROBBINS, T. W., & ZMIGROD, L. THE HABITUAL TENDENCIES QUESTIONNAIRE: A TOOL FOR PSYCHOMETRIC INDIVIDUAL DIFFERENCES RESEARCH. PERSONALITY AND MENTAL HEALTH. 2022;16(1):30-46.

ROGERS, L. Q., COURNEYA, K. S., VERHULST, S., ET AL. EXERCISE BARRIER AND TASK SELF-EFFICACY IN BREAST CANCER PATIENTS DURING TREATMENT. SUPPORTIVE CARE IN CANCER. 2006;14(1):84-90.

SALAM A, WOODMAN A, CHU A, ET AL. EFFECT OF POST-DIAGNOSIS EXERCISE ON DEPRESSION SYMPTOMS, PHYSICAL FUNCTIONING AND MORTALITY IN BREAST

CANCER SURVIVORS: A SYSTEMATIC REVIEW AND META-ANALYSIS OF RANDOMIZED CONTROL TRIALS. CANCER EPIDEMIOL. 2022;77:102-111.
SCHMIDT K, VOGT L, THIEL C, ET AL. VALIDITY OF THE SIX-MINUTE WALK TEST IN CANCER PATIENTS. INT J SPORTS MED. 2013;34(7):631-6.
TANGNEY, J. P., BAUMEISTER, R. F., & BOONE, A. L. HIGH SELF-CONTROL PREDICTS GOOD ADJUSTMENT, LESS PATHOLOGY, BETTER GRADES, AND INTERPERSONAL SUCCESS. JOURNAL OF PERSONALITY. 2004;72(2):271-324.
THANDI, G., FEAR, N. T., & CHALDER, T. A COMPARISON OF THE WORK AND SOCIAL ADJUSTMENT SCALE (WSAS) ACROSS DIFFERENT PATIENT POPULATIONS USING RASCH ANALYSIS AND EXPLORATORY FACTOR ANALYSIS. JOURNAL OF PSYCHOSOMATIC RESEARCH. 2017;92:45-48.
TWOMEY R, YEUNG ST, WRIGHTSON JG, ET AL. POST-EXERTIONAL MALAISE IN PEOPLE WITH CHRONIC CANCER-RELATED FATIGUE. J PAIN SYMPTOM MANAGE. 2020;60(2):407-416.
HANNA VAN WAART, MARTIJN M. STUIVER, WIM H. VAN HARTEN, ET AL. EFFECT OF LOW-INTENSITY PHYSICAL ACTIVITY AND MODERATE- TO HIGH-INTENSITY PHYSICAL EXERCISE DURING ADJUVANT CHEMOTHERAPY ON PHYSICAL FITNESS, FATIGUE, AND CHEMOTHERAPY COMPLETION RATES: RESULTS OF THE PACES RANDOMIZED CLINICAL TRIAL. JOURNAL OF CLINICAL ONCOLOGY. 2015;33(17):1918-1927.
VOOGT, E., VAN DER HEIDE, A., VAN LEEUWEN, A. F., ET AL. POSITIVE AND NEGATIVE AFFECT AFTER DIAGNOSIS OF ADVANCED CANCER. *PSYCHO-ONCOLOGY*; 2005, *14*(4): 262-273.
WATSON, D., CLARK, L. A., & TELLEGEN, A. DEVELOPMENT AND VALIDATION OF BRIEF MEASURES OF POSITIVE AND NEGATIVE AFFECT: THE PANAS SCALES. JOURNAL OF PERSONALITY AND SOCIAL PSYCHOLOGY; 1988, 54(6): 1063.
WEEMAES, A. T. R., SIEBEN, J. M., BEELEN, M., ET AL. DETERMINANTS OF PHYSICAL ACTIVITY MAINTENANCE AND THE ACCEPTABILITY OF A REMOTE COACHING INTERVENTION FOLLOWING SUPERVISED EXERCISE ONCOLOGY REHABILITATION: A QUALITATIVE STUDY. JOURNAL OF CANCER SURVIVORSHIP; 2023.
WEYLAND, S., FRITSCH, J., FEIL, K., ET AL. INVESTIGATING THE RELATION BETWEEN POSITIVE AFFECTIVE RESPONSES AND EXERCISE INSTIGATION HABITS IN AN AFFECT-BASED INTERVENTION FOR EXERCISE TRAINERS: A LONGITUDINAL FIELD STUDY. FRONTIERS IN PSYCHOLOGY. 2022;13.
ZHANG, X., LI, Y. & LIU, D. EFFECTS OF EXERCISE ON THE QUALITY OF LIFE IN BREAST CANCER PATIENTS: A SYSTEMATIC REVIEW OF RANDOMIZED CONTROLLED TRIALS. SUPPORT CARE CANCER. 2019;27, 9–21.

17 Appendices

1. **Participant information form and consent form**

2. **Advertisement**

3. **Questionnaires**

4. **Behaviour Change (Psychology) Questionnaires**
