# Supplementary material for: Evaluating the effect of the SMART intervention in people with recently diagnosed breast cancer who are being treated at a public tertiary hospital in Australia: protocol and statistical analysis plan for a single-blinded, single centre randomised controlled trial
Source: PLoS One. 2026 Jan 30;21(1):e0341423. doi: 10.1371/journal.pone.0341423 (PMC12857944; doi:10.1371/journal.pone.0341423)
Supplement: S4 File — (PDF) [file pone.0341423.s004.pdf]

Participant Information Sheet/Consent Form

Interventional Study - Adult providing own consent

Royal Perth Hospital

**Title**	The SMART exercise program for people recently diagnosed with breast cancer
**Short Title**	SMART exercise in breast cancer
**Protocol Number**	Version 5 (18th February, 2025)
**Coordinating Principal Investigator/ Principal Investigator**	Kylie Hill<br>Carol Watson
**Associate Investigator(s)**	Susan Stinton, Natasha Bear, Alejandro Dominguez Garcia, Dale Edwick, Sally Lynch, Adam Lloyd, Nola Mattingley, Chloe Maxwell-Smith, Niamh Moloney, Isabelle Simillion, Sheridan Simmons, Ru-Wen Teh, Yuin Lai, Shiv Meka, Suki Gill
**Location** (where CPI/PI will recruit)	Royal Perth Hospital, Wellington Street, Perth WA 6000<br>Sir Charles Gardner Hospital, Nedlands, WA 6009

Part 1 What does my participation involve?

1 Introduction

You are invited to take part in our research project because you are undergoing treatment for breast cancer (BC). The research project is trialling a new exercise program.

This Participant Information Sheet/Consent Form tells you about the research project. It explains the tests and treatments involved. Knowing what is involved will help you decide if you want to take part in the research.

Please read this information carefully. Ask questions about anything that you don't understand or want to know more about. Before deciding whether or not to take part, you might want to talk about it with a relative, friend or your local doctor.

Participation in this research is voluntary. If you don't wish to take part, you don't have to. You will receive the best possible care whether or not you take part.

If you decide you want to take part in the research project, you will be asked to sign the consent section. By signing it you are telling us that you:
• Understand what you have read
• Consent to take part in the research project
• Consent to have the tests and treatments that are described
• Consent to the use of your personal and health information as described.

You will be given a copy of this Participant Information and Consent Form to keep.

2 What is the purpose of this research?

Treatment for BC can affect your physical and emotional health which often results in time away from work and/or other meaningful life activities. One way you combat this is to ensure that you exercise regularly. Exercise is important for everyone's health and wellbeing. Although it might seem odd to start or increase exercise during cancer treatment, we know that exercise will help with your energy levels, physical function, emotional health and sleep. However, many people find it challenging to exercise during cancer treatments, so it is also important to have a health professional supporting and guiding you through this process.

As part of usual care at Royal Perth Hospital (RPH) Breast Clinic, you can see a physiotherapist to help you incorporate exercise into your daily life. However, as part of a study, we want to see if a more supported and individualised exercise program offers any greater benefit in terms of quality of life. A major part of this research is to have people tell us about their exercise and healthcare journey throughout their treatment. If this research shows that the more supported program improves care at the Breast Clinic at RPH, this will potentially change the way that exercise is offered.

This research has been funded by the RPH Research Foundation through the Vonesch Breast Cancer research grant.

This research is being conducted in conjunction with Curtin University.

3 What does participation in this research involve?

We are running this project over the next 2 years. You will be screened for eligibility to participate in this study. If you are eligible, you will be invited to participate in our project. If you would like to enrol to the study you will need to sign a consent form before being allocated to a study group.

This study will involve random allocation to one of two groups (group A or B). There is a one in two chance (50/50) you will be in group A or group B. Participants in both groups will need to attend the physiotherapy department at RPH for two assessments sessions (each taking about 2 hours). One will take place at the beginning of the study and the other wil take place about 16 weeks later. During these sessions, we will measure your strength, fitness and to fill out questionnaires relating to your health, work capacity and cancer-related side effects. You will also be asked to fill out questionnaires relating to your health and work 8 weeks after starting, and again twelve months after you first agreed to participate in the study.

If you are allocated to group A, you will receive usual care, and can see see a physiotherapist to help you incorporate exercise into your daily life. In addition, someone from the study will contact you over the phone/telehealth each month for 4 months to ask about how much exercise you have been doing.

If you are allocated to group B, in addition to usual care, you will be offered online and face-to-face exercise options over 16 weeks. During this time you will be assessed by a physiotherapist, provided with an exercise program which is individual to your exercise preferences, experience and goals. You will be required to attend the RPH gym and/or telehealth service weekly for exercise sessions and to help to support you through this process while you are undergoing your cancer treatment. Your exercise program will be varied depending on how well you are feeling.

We are doing this study because we do not know if the more supported and individualsd exercise program (i.e. group B) offers any greater benefit that usual care (i.e. group A) in terms of quality of life. To find out, we need to compare different treatments.

This research project has been designed to make sure the researchers interpret the results in a fair and appropriate way and avoids researcherss or participants jumping to conclusions.

There are no additional costs associated with participating in this research project, nor will you be paid. All medication, tests and medical care required as part of the research project will be provided to you free of charge.

You may be reimbursed for any reasonable travel and/or parking associated with the research project assessments to a maximum of a $15 voucher per session. You may also be provided with a $20 voucher to compensate you for the time it takes you to complete the assessments at each time point.

If you have a local doctor, we strongly recommend that you inform them of your participation in this research project.

4 What do I have to do?

You will need to have access to the internet so that you are able access information about your exercises and to fill in questionnaires.

Particpants in group A and B will do the same assessments. Those in group B will be offered support to exercise for 16 weeks.

You will also need to have access to a phone or computer to receive video or phone “telehealth” calls. These may be once a week, or once a month, depending on which group you are in.

You are able to continue with any other exercise that you may usually participate in if you would like to, but we will ask that you let us know about this.

You may also need to use a heart rate monitor (similar to a smart watch) while you are exercising. This will be provided to you.

There are no restrictions in terms of your diet or medication.

Most people are able to exercise safely while they are undergoing treatment for BC, but there are some cases where we may ask that you check with your doctor first.

5 Other relevant information about the research project

We are aiming to recruit 260 adults in this study through the Breast Clinic at RPH. There will be 130 people in group A and 130 in group B.

The researchers working on this project are based at Curtin University, the Physiotherapy Department, and the Breast Clinic at RPH.

6 Do I have to take part in this research project?

Participation in any research project is voluntary. If you do not wish to take part, you do not have to. If you decide to take part and later change your mind, you are free to withdraw from the project at any stage with reason or justification.

If you do decide to take part, you will be given this Participant Information and Consent Form to sign and you will be given a copy to keep.

Your decision whether to take part or not to take part, or to take part and then withdraw, will not affect your routine treatment, your relationship with those treating you or your relationship with RPH.

7 What are the alternatives to participation?

You do not have to take part in this research project to receive treatment at this hospital. Other options are available; these include usual physiotherapy care. The researchers will discuss these options with you before you decide whether or not to take part in this research project. You can also discuss the options with your specialist or local doctor.

8 What are the possible benefits of taking part?

We cannot guarantee or promise that you will receive any benefits from this research; however, possible benefits may include direct physical and emotional health benefits. In addition, this study will provide a better understanding how best to support people with BC to exercise during their treatment. This may improve future care for other adults with BC.

9 What are the possible risks and disadvantages of taking part?

Medical treatments often cause side effects. You may have none, some or all of the effects listed below, and they may be mild, moderate or severe. If you have any of these side effects, or are worried about them, talk with your researcher. Your researcher will also be looking out for side effects.

There may be side effects that the researchers do not expect or do not know about and that may be serious. Tell your researcher immediately about any new or unusual symptoms that you get.

Many side effects go away shortly after treatment ends. However, sometimes side effects can be serious, long lasting or permanent. If a severe side effect or reaction occurs, your researcher may need to stop your treatment. Your researcher will discuss the best way of managing any side effects with you.

The most likely side effects include:

- Muscle soreness
- A severe type of tiredness felt by some adults with cancer, called Post Exertional Malaise (PEM)

If you participate in this study we will monitor both of these potential side effects. If they bother you then your exercise program can be eased off so that either of these possible symptoms are manageable for you. Neither of these side effects require treatment.

Side Effect	How often is it likely to occur?	How severe might it be?	How long might it last?
Muscle soreness	Likely to occur with harder exercise sessions.	Muscle soreness after exercise is usually mild. You should still be able to carry out your usual daily activities	Muscle soreness usually peaks 1 or 2 days after exercise. It usually only lasts for a day or so.
Post exertional malaise (PEM)	Mild fatigue after exercise is common. More severe fatigue or PEM is less likely.	Increasing fatigue after exercise is usually mild. You should be able to carry out your usual activities. PEM is a more severe form of fatigue and may mean you want to stay in bed for a day after exercising.	PEM usually lasts a day or so after exercise.

If participation in this research uncovers a medical condition of which you were unaware, your physiotherapist will refer you back to your usual GP or oncology specialist.

When completing the questionnaires, you may feel that some of the questions are stressful or upsetting. If you do not wish to answer a question, you may skip it and go to the next question,

have a break, or stop completing the questionnaire. If you become upset or worried due to your participation in this research project, you may reach out to the researchers involved in this project (details at the end of this document) or contact your Breast Cancer Nurse, specialist or GP, or relevant health professional to seek internal referral guidance. If you would like to seek support from external sources, you may wish to contact the following services:

Breast Cancer Care WA – (08) 9324 3703 (Monday to Friday 8.30am – 4.30pm)
Breast Cancer Network Australia – 1800 500 258 (Monday to Friday 9.00am – 5.00pm)
Lifeline – 13 11 14 (Available 24/7)
Beyond Blue – 1300 224 636 (Available 24/7)

10 What if new information arises during this research project?

Sometimes during the course of a research project, new information becomes available about the treatment that is being studied. If this happens, your researcher will tell you about it and discuss with you whether you want to continue in the research project. If you decide to withdraw, your researcher will make arrangements for your regular health care to continue. If you decide to continue in the research project you will be asked to sign an updated consent form.

Also, on receiving new information, your researcher might consider it to be in your best interests to withdraw you from the research project. If this happens, they will explain the reasons and arrange for your regular health care to continue.

11 Can I have other treatments during this research project?

This trial will not impact medication use.

12 What if I withdraw from this research project?

If you decide to withdraw from the project, please notify a member of the research team before you withdraw. This notice will allow that person or the research supervisor to discuss any health risks or special requirements linked to withdrawing.

If you do withdraw your consent during the research project, the researcher and relevant study staff will not collect additional personal information from you, although personal information already collected will be retained to ensure that the results of the research project can be measured properly and to comply with law. You should be aware that data collected by the researchers up to the time you withdraw will form part of the research project results. If you do not want them to do this, you can tell them when you join the research project, or when you withdraw from the study.

13 Could this research project be stopped unexpectedly?

This research project may be stopped unexpectedly for a variety of reasons. These may include reasons such as:

- Unacceptable side effects
- The treatment being shown not to be effective
- The treatment being shown to work and not need further testing

14 What happens when the research project ends?

For those in Group A, the supported exercise program will run for 16 weeks. For those in Group B, the monthly phone calls will also finish after 16 weeks. At this point, all participants (group A and B) you will be required to attend an assessment in the physiotherapy department at RPH (in

person). Once you have completed the program, your physiotherapist will discuss ongoing options in the community so that you are able to continue to exercise regularly.

Eight months after you have completed your program, the researchers will contact you so that you can fill out questionnaires relating to your general health and the time you took off work during your BC treatment.

Once the study has been completed (at the end of 2026), overall results from the study will be available by contacting the study team.

Part 2 How is the research project being conducted?

15 What will happen to information about me?

By signing the consent form you consent to the research team collecting and using personal information about you for the research project. Any information obtained in connection with this research project that can identify you will remain confidential.

Data collected for to this study will use a computer program called REDCap software (licenced to Curtin University). After informed consent is given, participants will be given a study number. This will be used in lieu of any identifying information in REDCap and when the data is exported from REDCap. All data that is downloaded from this software will be kept on a secure, password protected server at RPH. Any identifying information will be stored on the secure network drive at RPH, only accessible by study researchers who work at WA Health. This information will only be available to researchers working on this project. REDCap users have all activity tracked. Log on to the software requires all users to verify their identity using authenticator codes. Data will be stored for 7 years. Following this time, electronic files will be deleted. Your information will only be used for the purpose of this research project and it will only be disclosed with your permission, except as required by law.

Your health records will be accessed, and the relevant information accessed will form part of the study data. By signing the consent form you agree to the study team accessing health records if they are relevant to your participation in this research project.

It is anticipated that the results of this research project will be published and/or presented in a variety of forums. In any publication and/or presentation, information will be provided in such a way that you cannot be identified, except with your permission. Only data relating to the study group (rather than individuals) will be reported.

Information about your participation in this research project may be recorded in your health records.

In accordance with relevant Australian and West Australian privacy and other relevant laws, you have the right to request access to your information collected and stored by the research team. You also have the right to request that any information with which you disagree be corrected. Please contact the study team member named at the end of this document if you would like to access your information.

Any information obtained for the purpose of this research project that can identify you will be treated as confidential as far as the law allows, and securely stored. It will be disclosed only with your permission, or as required by law.

16 Complaints and compensation

If you have any complaints relating to the study please contact Kylie Hill (k.hill@curtin.edu.au) or the contacts below at any time point.

If you suffer any injuries or complications as a result of this research project, you should contact the study team as soon as possible and you will be assisted with arranging appropriate medical treatment. If you are eligible for Medicare, you can receive any medical treatment required to treat the injury or complication, free of charge, as a public patient in any Australian public hospital.

17 Who is organising and funding the research?

This research project is being conducted by The RPH Research Foundation in conjunction with Curtin University.

This research is being funded through a research grant called the *Vonesch Breast Cancer Rehabilitation Grant*.

18 Who has reviewed the research project?

All research in Australia involving humans is reviewed by an independent group of people called a Human Research Ethics Committee (HREC). The ethical aspects of this research project have been approved by the WA Health Central HREC..

This project will be carried out according to the *National Statement on Ethical Conduct in Human Research (2007)*. This statement has been developed to protect the interests of people who agree to participate in human research studies.

19 Further information and who to contact

The person you may need to contact will depend on the nature of your query.

If you want any further information concerning this project or if you have any medical problems which may be related to your involvement in the project (for example, any side effects), you can contact the principal study researcher on 9224 2076 or any of the following people:

Clinical contact person

Name	Susan Stinton
Position	Researcher and Physiotherapist<br>Royal Perth Hospital Physiotherapy Department
Telephone	9224 2076
Email	susan.stinton2@health.wa.gov.au

For matters relating to research at the site at which you are participating, the details of the local site complaints person are:

Complaints contact person

Name	Central Office for Research Ethics (CORE)
Position	Research Governance Coordinator
Telephone	08 9222 4214
Email	HREC@health.wa.gov.au

This project has been granted ethical approval by the
WA Health Central *Human Research Ethics Committee (HREC)*. The ethics approval number (RGS0000006136).

Consent Form - *Adult providing own consent*

**Title**	The SMART exercise program for people recently diagnosed with breast cancer
**Short Title**	SMART exercise in breast cancer
**Protocol Number**	Version 5.0 (18th February 2025)
**Coordinating Principal Investigator**	Kylie Hill
**Principal Investigator**	Carol Watson
**Associate Investigator(s)**	Susan Stinton, Natasha Bear, Alejandro Dominguez Garcia, Dale Edwick, Sally Lynch, Adam Lloyd, Nola Mattingley, Chloe Maxwell-Smith, Niamh Moloney, Isabelle Simillion, Sheridan Simmons, Ru-Wen Teh, Yuin Lai, Shiv Meka, Suki Gill
**Location** (where CPI/PI will recruit)	Royal Perth Hospital, Wellington Street, Perth, WA 6000<br>Sir Charles Gardner Hospital, Nedlands, WA 6009

Declaration by Participant

I have read the Participant Information Sheet or someone has read it to me in a language that I understand. I understand the purposes, procedures and risks of the research described in the project.

I have had an opportunity to ask questions and I am satisfied with the answers I have received.

I freely agree to participate in this research project as described and understand that I am free to withdraw at any time during the study without affecting my future health care.

☐ I consent for my name, phone or email to be used on *Physitrack* and the Fitbit program so that I can access an online version of my exercise program and so the researchers can view the activity and heart rate information from the Fitbit.

I understand that I will be given a signed copy of this document to keep.

Name of Participant (please print) ______________________________

Signature ______________________ Date ______________________

Name of Witness* to participant's Signature (please print) ______________________

Signature ______________________ Date ______________________

* Witness is not to be the investigator, a member of the study team or their delegate. In the event that an interpreter is used, the interpreter may not act as a witness to the consent process. Witness must be 18 years or older.

Declaration by Study Clinician /Senior Researcher† I have given a verbal explanation of the research project, its procedures and risks and I believe that the participant has understood that explanation.

Name of Study Clinician /
Senior Researcher† (please print) ______________________________

Signature ______________________ Date ______________________

† A senior member of the research team must provide the explanation of, and information concerning, the research project. Note: All parties signing the consent section must date their own signature.

Form for Withdrawal of Participation - *Adult providing own consent*

**Title**	The SMART exercise program for people recently diagnosed with breast cancer
**Short Title**	SMART exercise in breast cancer
**Protocol Number**	Version 5.0 (18th February 2025)
**Coordinating Principal Investigator**	Kylie Hill
**Principal Investigator**	Carol Watson
**Associate Investigator(s)**	Susan Stinton, Natasha Bear, Alejandro Dominguez Garcia, Dale Edwick, Sally Lynch, Adam Lloyd, Nola Mattingley, Chloe Maxwell-Smith, Niamh Moloney, Isabelle Simillion, Sheridan Simmons, Ru-Wen Teh
**Location** (where CPI/PI will recruit)	Royal Perth Hospital, Wellington Street, Perth WA 6000<br>Sir Charles Gardner Hospital, Nedlands, WA 6009

Declaration by Participant

I wish to withdraw from participation in the above research project and understand that such withdrawal will not affect my routine treatment, my relationship with those treating me or my relationship with Royal Perth Hospital.

Please tick (✓) the appropriate box below:

☐ I wish to withdraw from participation in this study, and agree to the continued use of my data collected to this point

☐ I wish to withdraw from participation in this study, I also wish to withdraw my data collected up to this point.

Name of Participant (please print) ______________________________

Signature ______________________ Date ______________________

In the event that the participant's decision to withdraw is communicated verbally, the Study Clinician/Senior Researcher will need to provide a description of the circumstances below.

Declaration by Study Clinician/Senior Researcher†

I have given a verbal explanation of the implications of withdrawal from the research project and I believe that the participant has understood that explanation.

Name of Study Clinician / Senior Researcher† (please print) ______________________________

Signature ______________________ Date ______________________

† A senior member of the research team must provide the explanation of and information concerning withdrawal from the research project. Note: All parties signing the consent section must date their own signature.
