# Supplementary material for: Evaluating the effect of the SMART intervention in people with recently diagnosed breast cancer who are being treated at a public tertiary hospital in Australia: protocol and statistical analysis plan for a single-blinded, single centre randomised controlled trial
Source: PLoS One. 2026 Jan 30;21(1):e0341423. doi: 10.1371/journal.pone.0341423 (PMC12857944; doi:10.1371/journal.pone.0341423)
Supplement: S5 File — (PDF) [file pone.0341423.s005.pdf]

PARTICIPANT NUMBER: ______________________
DATE: ____________________________________

HEALTHCARE UTILISATION

The following survey is about any health care appointments and services you have used recently.

We would like to know which health care providers you have consulted in the past 2 months. It is about consultations for yourself.

Which consultations count?
- **Visits to a health care provider at RPH or anywhere else**
- **Appointments because you had a physical or psychological complaint**
- **Appointments where the health care provider came to your home**
- **Telephone appointments**
- **Phone calls Which consultations do not count?**
- **Appointments for another person, for example for your child**
- **Telephone calls to make an appointment**

Are you unsure about the exact number of consultations? Please fill in the approximate number of consultations you have had.

1a. Have you consulted a general practitioner (GP) in the past 2 months?
- ☐ No
- ☐ Yes ____________________________________ *(specify number of appointments)*

____

1b. Have you seen a nurse (as an outpatient) in the last 2 months?
- ☐ No
- ☐ Yes ____________________________________ *(specify number of appointments)*

____

2. Have you seen a social worker in the last 2 months?
- ☐ No
- ☐ Yes ____________________________________ *(specify number of appointments)*

____

3. Have you seen a physiotherapist in the last 2 months?
- ☐ No
- ☐ Yes ____________________________________ *(specify number of appointments)*

____

4. Have you seen an occupational therapist in the last 2 months?
- ☐ No
- ☐ Yes ____________________________________ *(specify number of appointments)*

____

5. Have you seen a speech therapist in the last 2 months?
- ☐ No
- ☐ Yes ____________________________________ *(specify number of appointments)*

____

PARTICIPANT NUMBER: ____________________
DATE: ________________________________

---

6. Have you seen a dietitian in the last 2 months?
   - ☐ No
   - ☐ Yes ________________________________ *(specify number of appointments)*

---

7. Have you seen another allied health or medical practitioner(s) in the past 2 months, including an acupuncturist, Chinese medicine practitioner, naturopath, chiropractor and/or osteopath?
   - ☐ No
   - ☐ Yes ________________________________ *(total number of appointments)*

---

8. Have you seen a psychologist in the last 2 months?
   - ☐ No
   - ☐ Yes ________________________________ *(specify number of appointments)*

---

9. Have you seen a podiatrist in the last 2 months?
   - ☐ No
   - ☐ Yes ________________________________ *(specify number of appointments)*

---

10. Have you seen an exercise physiologist in the last 2 months?
   - ☐ No
   - ☐ Yes ________________________________ *(specify number of appointments)*

---

11. Have you seen a dentist in the last 2 months?
   - ☐ No
   - ☐ Yes ________________________________ *(specify number of appointments)*

---

12. Have you seen an Aboriginal and Torres Strait Islander Health Practitioner in the last 2 months?
   - ☐ No
   - ☐ Yes ________________________________ *(specify number of appointments)*

---

13. Have you had any appointments to perform any medical imaging in the last 2 months?
   e.g. for an x-ray, Yes ultrasound, CT scan or other type of imaging
   - ☐ No
   - ☐ Yes ________________________________ *(specify number of appointments)*

---

14. Did you have any appointments with your employment or workplace company doctor in the past 2 months?
   - ☐ Not applicable
   - ☐ No
   - ☐ Yes ________________________________ *(specify number of appointments)*

PARTICIPANT NUMBER: ______________________
DATE: _____________________________________

---

15. Have you received home care in the last 2 months?
- ☐ No (*go to question 16)*
- ☐ Yes ____________________________________

What kind of home care have you had in the past 2 months?
You can tick more than 1 box
- ☐ Housekeeping/domestic help (example: vacuuming, making bed, going for daily groceries)
- ☐ Personal care (example: help with bathing or dressing)
- ☐ Nursing (example: putting on a bandage, administering medication

How many weeks did you have this home care?
Count up all weeks in the past 2 months. Note: a period of 2 months counts as 8 weeks
Domestic help: ______ weeks in the last 2 month
Personal care: ______ weeks in the last 2 months
Nursing: ______ weeks in the last 2 months

How many hours of home care did you receive on average in these weeks?
Domestic help: ______ average hours a week
Personal care: ______ average hours a week
Nursing: ______ average hours a week

---

16. What medications did you take in the past 2 months?
By medication we mean all drugs that you have received on prescription and medication that you have bought at the pharmacy or drugstore. There are three examples below.

Pay attention: look at the package! It shows how much you had to take each time and how often you had to do so per day. Have you used more or less? Then enter how much you actually used.

What is the name of the medication?	How much did you take each time?	How many times did you take this each day?	On how many days in the past 2 months have you used this medication?
Example 1<br>*Metroprolol*	*Example 1*<br>*100mg*	*Example 1*<br>*1 time*	*Example 1*<br>*90 days*
Example 2<br>*Furosemide (diuretic)*	*Example 2*<br>*40mg*	*Example 2*<br>*1 time*	*Example 2*<br>*16 days (2x per week)*
Example 3<br>*Hydrocortisone cream*	*Example 3*<br>*-*	*Example 3*<br>*1*	*Example 3*<br>*14 days*

PARTICIPANT NUMBER: ____________________
DATE: ________________________________

---

17. Did you visit the emergency room of a hospital in the past 2 months?
    - ☐ No
    - ☐ Yes ________________________________ *(specify number of appointments)*

---

18. Have you been taken to the hospital in an ambulance in the past 2 months?
    - ☐ No
    - ☐ Yes ________________________________ *(specify number of appointments)*

---

19. Did you have an appointment at a medical outpatient clinic of a hospital in the last 2 months?
For example, with a cardiologist, oncologist or neurologist.
    - ☐ No
    - ☐ Yes ________________________________ *(specify number of appointments)*

    What type of doctor did you visit in the hospital clinic?
    1 _________________________________
    2 _________________________________
    3 _________________________________
    4 _________________________________
    5 _________________________________
    6 _________________________________

---

20. Did you visit the hospital for day care treatment during the past 2 months?
i.e. you did not stay overnight. Examples of day care treatments are blood transfusions, renal dialysis or chemo at RPH or any other hospital.
    - ☐ No
    - ☐ Yes

    For what kind of treatment was this? .
    If you had more than 6, please enter additional types in the comments section at the bottom of this questionnaire.

    Treatment 1: _________________________________
    Treatment 2: _________________________________
    Treatment 3: _________________________________
    Treatment 4: _________________________________
    Treatment 5: _________________________________
    Treatment 6: _________________________________

    How many times did you have to go to the hospital for these treatments in the past 3 months?
    e.g. to RPH or any other hospital

    _____________________________________ (total number of treatments)

PARTICIPANT NUMBER: ______________________
DATE: ____________________________________

---

21. Did you go elsewhere for day care treatment in the past 2 months?
i.e. you did not stay overnight. For example, you went to the day care treatment centre of a residential/care centre, a psychiatric institution, or a rehabilitation centre

☐ No
☐ Yes

What kind of institution was this?
You can tick more than 1 box

☐ Residential care centre or nursing home
☐ Rehabilitation canre
☐ Mental health institution
☐ Other (specify: ______________________________________)

How many times did you have to go here in the past 2 months? ______________________________
Enter the total number of times you have been to all of these institutions.

---

22. Have you been admitted to a hospital for inpatient care in the past 2 months?
i.e. you had to stay overnight. For example, because you had surgery and could not go home immediately. Included RPH and any other hospital

☐ No
☐ Yes ___________________________________ *(times in the last 2 months)*

---

23. Have you been admitted elsewhere for your health in the past 2 months?
For example, in a residential care centre, psychiatric institution or rehabilitation centre

☐ No
☐ Yes

What kind of institution was this?

☐ Residential care centre or nursing home
☐ Rehabilitation centre
☐ Mental health institution
☐ Other (specify: ______________________
