# Supplementary material for: Evaluating the effect of the SMART intervention in people with recently diagnosed breast cancer who are being treated at a public tertiary hospital in Australia: protocol and statistical analysis plan for a single-blinded, single centre randomised controlled trial
Source: PLoS One. 2026 Jan 30;21(1):e0341423. doi: 10.1371/journal.pone.0341423 (PMC12857944; doi:10.1371/journal.pone.0341423)
Supplement: Attachment — Submitted filename: Response to reviewers.docx [file pone.0341423.s007.docx]

9th September 2025

Dear Dr Khan,

Thank you for your feedback and the suggestions from yourself and the two reviewers. We appreciate the opportunity to re-submit our paper and hope you find these changes acceptable.

Included in our replies below is a response regarding the financial disclosure statement (see *Comments 2* and *3*). We confirm we are happy for you to amend this in the online form, with our thanks. The exact wording for the full funding statement is specified under the reply to *Comment 3*.

We also note the requirement to disclose any changes to the reference list in this letter. These changes have been outlined in *Comment 7* and *Comment 9* below.

The following statements and sections have been checked and revised as follows:

**Comment 1: Regarding manuscript style**

*Please ensure that your manuscript meets PLOS ONE's style requirements, including those for file naming.*

Thank you, we have double checked the manuscript to ensure it is in line with PLOS ONE style requirements.

**Comment 2: regarding the financial disclosure**

*We note that one or more of the authors is affiliated with the funding organization, indicating the funder may have had some role in the design, data collection, analysis or preparation of your manuscript for publication; in other words, the funder played an indirect role through the participation of the co-authors. If the funding organization did not play a role in the study design, data collection and analysis, decision to publish, or preparation of the manuscript and only provided financial support in the form of authors' salaries and/or research materials, please do the following:*

*1. Review your statements relating to the author contributions, and ensure you have specifically and accurately indicated the role(s) that these authors had in your study. These amendments should be made in the online form.*

One author, Susan Stinton, has her salary provided by the funder. This is disclosed in the funding statement as below.

*2. Confirm in your cover letter that you agree with the following statement, and we will change the online submission form on your behalf:*

Thank you for changing this online submission form on our behalf, we confirm we are happy with the following wording for this paragraph in the funding statement:

[The funder provided peer review of this protocol design and support in the form of salary for one author [SS], but did not have any additional role in the study design, data collection and analysis, decision to publish, or preparation of the manuscript. The specific roles of this author are articulated in the ‘author contributions’ section. https://www.rphresearchfoundation.org.au/]

**Comment 3: Acknowledgements section**

*We note that you have provided funding information that is currently declared in your Funding Statement. However, funding information should not appear in the Acknowledgments section or other areas of your manuscript. We will only publish funding information present in the Funding Statement section of the online submission form.*

*Please remove any funding-related text from the manuscript and let us know how you would like to update your Funding Statement.*

Apologies for this oversight. The manuscript has been checked to ensure there is no funding information in-text, and the correct wording for the Acknowledgement statement in the manuscript has been updated as follows:

[In-kind support from the Physiotherapy Department at RPH has enabled the development and implementation of this study. One of the authors (ADG) is supported by an Australian Government Research Training Program Scholarship through Curtin University. We would also like to thank the staff in the Breast Clinic, Medical Oncology Clinics and Physiotherapy Department at Royal Perth Hospital, and the team at Sir Charles Gardner Hospital Radiation Oncology team for their ongoing involvement with this study.]

The wording for the Funding Statement (consistent with *Comment 2* above) is as follows:

[This research is funded by the Vonesch Breast Cancer Rehabilitation Grant (S01/2023), through the RPH Research Foundation. This funding was made possible from a gift in Memory of Felix Viktor Vonesch and Verena Vonesch.

Funding acquisition: KH, AL, CW

The funder provided peer review of this protocol design and support in the form of salary for one author [SS], but did not have any additional role in the study design, data collection and analysis, decision to publish, or preparation of the manuscript. The specific roles of this author are articulated in the ‘author contributions’ section. <https://www.rphresearchfoundation.org.au/>]

**Comment 4: regarding data availability statement**

*When completing the data availability statement of the submission form, you indicated that you will make your data available on acceptance. We strongly recommend all authors decide on a data sharing plan before acceptance, as the process can be lengthy and hold up publication timelines. Please note that, though access restrictions are acceptable now, your entire data will need to be made freely accessible if your manuscript is accepted for publication. This policy applies to all data except where public deposition would breach compliance with the protocol approved by your research ethics board. If you are unable to adhere to our open data policy, please kindly revise your statement to explain your reasoning and we will seek the editor's input on an exemption. Please be assured that, once you have provided your new statement, the assessment of your exemption will not hold up the peer review process.*

Thank you for clarifying this requirement. As this current paper under review is a study protocol, there is no associated data to share. We accept and acknowledge that future publications will require data sharing. Please let us know if you wish to address this in a different way.

The revised data sharing statement is as follows:

[This is a protocol paper, therefore no datasets were generated or analysed. We accept and acknowledge that future publications will require data sharing. Full data management procedures are detailed in the full protocol, Supplementary Item 2]

**Comment 5: also regarding data availability**

*In the online submission form, you indicated that [This is a protocol paper, no datasets were generated or analysed. Upon study completion, the data generated from this study will be available from an online repository or from the corresponding author upon reasonable request, as far as possible under legislation. Full data management procedures are detailed in the full protocol, Supplementary Item 2.]. All PLOS journals now require all data underlying the findings described in their manuscript to be freely available to other researchers, either 1. In a public repository, 2. Within the manuscript itself, or 3. Uploaded as supplementary information. This policy applies to all data except where public deposition would breach compliance with the protocol approved by your research ethics board. If your data cannot be made publicly available for ethical or legal reasons (e.g., public availability would compromise patient privacy), please explain your reasons on resubmission and your exemption request will be escalated for approval.*

As per above in *Comment 4*, please note that this is a study protocol, there is no associated data to share. We accept and acknowledge that future publications will require data sharing. Please let us know if you would like us to address this in a different way.

**Comment 6: regarding the author list**

*One of the noted authors is a group or consortium [SMART Study Team]. In addition to naming the author group, please list the individual authors and affiliations within this group in the acknowledgments section of your manuscript. Please also indicate clearly a lead author for this group along with a contact email address.*

Apologies for this confusion. The [SMART Study Team] was referred to in the cover letter only. All individual members of this team are listed on the title page and on the online submission.

**Comment 7: Reference list**
*Please review your reference list to ensure that it is complete and correct. If you have cited papers that have been retracted, please include the rationale for doing so in the manuscript text, or remove these references and replace them with relevant current references. Any changes to the reference list should be mentioned in the rebuttal letter that accompanies your revised manuscript. If you need to cite a retracted article, indicate the article’s retracted status in the References list and also include a citation and full reference for the retraction notice.*

Noted, thank you. Please refer to the answer in *Comment 9* regarding changes to the reference list.

In addition to the above requests regarding the statements and article sections we have addressed your additional comments. Thank you for these suggestions, they have been reviewed and revised as follows:

**Comment 8: regarding the title**

*Title: In the title, mention study settings and design, i.e., Self-administered dual-task training reduces balance deficits and falls among community-dwelling older adults: a multicentre parallel-group randomised controlled trial with economic evaluation protocol*

To incorporate the settings and design, the title has been amended

[Evaluating the effect of the SMART intervention in people with recently diagnosed breast cancer who are being treated at a public tertiary hospital in Australia: protocol and statistical analysis plan for a randomised controlled trial]

Comment 9: Also regarding the references

*For references, please update all citations to include only those from the last five years in the introduction. Use a current cohort of references that is no older than five years.*

Thank you, we have updated our reference list accordingly. All of the references in the introduction are no older than five years. However, we have kept references older than five years in the remainder of the manuscript, where they are original references relating to disease classification systems, original descriptions of outcome assessments, and other core references relating to current national treatment guidelines i.e. #17-19, 21-22, 24, 26, 33, 35, 37- 41, 43, 44-48.

We have done this to give credit where credit is due. Please let us know if you would like us to address this in a different way.

*Additionally, please avoid using multiple references unnecessarily; try to limit to one or, at most, two references.*

These have been revised to only include the most relevant 1-2 references as appropriate.

The changes to address the above comments regarding the age and use of multiple references have resulted in changes to the reference list.

The following references have been removed:

1. Brown JC, Huedo-Medina TB, Pescatello LS, Ryan SM, Pescatello SM, Moker E, et al. The efficacy of exercise in reducing depressive symptoms among cancer survivors: a meta-analysis. PLoS One. 2012; 7(1):e30955. doi:0.1371/journal.pone.0030955.
2. Ferreira AR, Di Meglio A, Pistilli B, Gbenou AS, El-Mouhebb M, Dauchy S, et al. Differential impact of endocrine therapy and chemotherapy on quality of life of breast cancer survivors: a prospective patient-reported outcomes analysis. Ann Oncol. 2019; 30(11):1784-95. doi: 10.1093/annonc/mdz298.
3. Campbell KL, Winters-Stone KM, Wiskemann J, May AM, Schwartz AL, Courneya KS, et al. Exercise Guidelines for Cancer Survivors: Consensus Statement from International Multidisciplinary Roundtable. Med Sci Sports Exerc. 2019; 51(11):2375-90. doi:10.1249/MSS.0000000000002116.
4. Cormie P, Atkinson M, Bucci L, Cust A, Eakin E, Hayes S, et al. Clinical Oncology Society of Australia position statement on exercise in cancer care. Med J Aust. 2018;209(4):184-7. doi: 10.5694/mja18.00199.
5. Segal R, Zwaal C, Green E, Tomasone J, Loblaw A, Petrella T. Exercise for people with cancer: a clinical practice guideline. Current oncology. 2017; 24(1):40-6. doi:10.3747/co.24.3376.
6. Spence R, Sandler C, Newton R, Galvão D, Hayes S. Physical Activity and Exercise Guidelines for People With Cancer: Why Are They Needed, Who Should Use Them, and When? Seminars in Oncology Nursing. 2020; 36(5). doi:https://doi.org/https://doi.org/10.1016/j.soncn.2020.151075
7. Mishra SI, Scherer RW, Geigle PM, Berlanstein DR, Topaloglu O, Gotay CC, et al. Exercise interventions on health-related quality of life for cancer survivors. Cochrane Database Syst Rev. 2012; 2012(8):CD007566. doi:10.1002/14651858.CD007566.pub2.
8. Zhang X, Li Y, Liu D. Effects of exercise on the quality of life in breast cancer patients: a systematic review of randomized controlled trials. Support Care Cancer. 2019; 27(1):9-21. doi: 10.1007/s00520-018-4363-2.
9. Meneses-Echavez JF, Gonzalez-Jimenez E, Ramirez-Velez R. Effects of supervised exercise on cancer-related fatigue in breast cancer survivors: a systematic review and meta analysis. BMC Cancer. 2015; 15:77. doi: 10.1186/s12885-015-1069-4.
10. Van Waart H, Stuiver MM, van Harten 521 WH, Geleijn E, Kieffer JM, Buffart LM, et al. Effect of Low-Intensity Physical Activity and Moderate- to High-Intensity Physical Exercise During Adjuvant Chemotherapy on Physical Fitness, Fatigue, and Chemotherapy Completion Rates: Results of the PACES Randomized Clinical Trial. J Clin Oncol. 2015; 33(17):1918-27.525 doi: 10.1200/JCO.2014.59.1081.
11. Hardcastle S, Maxwell-Smith C, Kamarova S, Lamb S, Millar L, Cohen P. Factors influencing non-participation in an exercise program and attitudes towards physical activity amongst cancer survivors. Supportive Care in Cancer. 2018; 26:1289-95. doi;26:1289-95.

The following references have been added:

1. Di Meglio A, Havas J, Gbenou AS, Martin E, El-Mouhebb M, Pistilli B, et al. Dynamics of Long-Term Patient-Reported Quality of Life and Health Behaviors After Adjuvant Breast Cancer Chemotherapy. J Clin Oncol. 2022;40(27):3190–204.
2. Hart NH, Stout NL, Haywood D, Ashbury FD, Chan RJ, Fitch MI, et al. World Health Organization package of interventions for rehabilitation for cancer: a MASCC‑endorsed resource for global action to address unmet rehabilitation needs of people affected by cancer. Supportive Care in Cancer. 2024;32(7):417.
3. Cormie P, Atkinson M, Bucci L, Cust A, Eakin E, Hayes S, et al. Clinical Oncology Society of Australia position statement on exercise in cancer care (Version 3). Med J Australia. 2020;209(4):184–7.
4. Tian E, Wilson O, Wojcik K, Butera G, Schneider J, Rogers L, et al. Recreational physical activity and health-related quality of life among breast cancer survivors: a systematic review. Qual Life Res. 2025;Jul 2.
5. Nie X, Yang T, Nie X, Yuan J. Comparative effects of different types of physical activity on health-related quality of life in breast cancer survivors: A systematic review, network meta-analysis, and meta-regression. Heliyon. 2024;10(10):e31555.
6. Zhou R, Chen Z, Zhang S, Wang Y, Zhang C, Lv Y, et al. Effects of Exercise on Cancer-Related Fatigue in Breast Cancer Patients: A Systematic Review and Meta-Analysis of Randomized Controlled Trials. Life (Basel). 2024;14(8).
7. Walker RC, Pezeshki P, Barman S, Ngan S, Whyte G, Lagergren J, et al. Exercise During Chemotherapy for Cancer: A Systematic Review. J Surg Oncol. 2024;130(8):1725–36.
8. Craig CL, Marshall AL, Sjostrom M, Bauman AE, Booth ML, Ainsworth BE, et al. International physical activity questionnaire: 12-country reliability and validity. Med Sci Sports Exerc. 2003;35(8):1381–95.
9. Bourke S, Bennett B, Oluboyede Y, Li T, Longworth L, O'Sullivan SB, et al. Estimating the minimally important difference for the EQ-5D-5L and EORTC QLQ-C30 in cancer. Health Qual Life Outcomes. 2024;22(1):81.
10. Wang J, Tan S, Gianotti L, Wu G. Evaluation and management of body composition changes in cancer patients. Nutrition. 2023;114:112132.
11. Leach HJ, Fairman CM, Gomes EL, Marker RJ. Evaluating individual level change in physical function response following an exercise program for cancer survivors. Support Care Cancer. 2023;31(6):353.
12. Tangney JP, Baumeister RF, Boone AL. High self-control predicts good adjustment, less pathology, better grades, and interpersonal success. J Pers. 2004;72(2):271–324.
13. Wijesuriya R, Moreno‐Betancur, M., Carlin, J. B., White, I. R., Quartagno, M., & Lee, K. J. . Multiple imputation for longitudinal data: A tutorial. Statistics in Medicine. 2025;44:3–4.

**Comment 10: lines 85 to 88 (of original manuscript)**

*Explain why you chose the SMART intervention. What are the differences from other interventions, like Otego exercise and a self-administered dual task?*

Thank you for suggesting this. We have added a section at the start of this paragraph on line 84:

[To optimise exercise among adults undergoing treatment for breast cancer, an exercise intervention was co-designed by a team that included physiotherapists, oncologists, psychologists, exercise physiologists and consumer representatives with lived experience of breast cancer. This exercise intervention has several novel aspects such as; (i) bespoke tailoring of the program which considers age, baseline fitness levels, individual preferences and breast cancer stage, (ii) inclusion and detailed mapping of behaviour change techniques, and (iii) close monitoring using wearable devices to allow modification of programs dependent on treatment and other relevant health symptoms.]

**Comment 11: In lines 90 to 96 (of original manuscript)**

*Rewrite the following standard manuscript.*

Apologies, we are unsure of what is meant by this request.

If referring to the structure of the study aim, we have followed the standard PICO format. We are happy to revise this section if there is preference for an alternate structure?

**Comment 12: In lines 98 to 105 (of original manuscript)**

*Explain why you want to “explore” (line 98) …., “investigate” (line 101) …and “assess” (line 103)…. From “explore, investigate, and assess,” who will benefit and how?*

Thank you, this paragraph has been revised and updated in the manuscript (from line 105 of the revised manuscript):

[In addition, we will explore variables that influence the magnitude of any change in HRQoL following completion of the SMART intervention. These potential moderators include treatment pathways, cancer stage, and the type, quantity and frequency of exercise undertaken. Understanding the influence of these variables may help to elucidate factors that modify the magnitude of between-group differences which will inform future service delivery and target those most likely to benefit. Additionally, we will investigate whether psychological determinants of behaviour change, such as capability, opportunity, and motivation, predict changes in physical activity levels from baseline to post-intervention and to long-term follow-up. This will help identify which behavioural determinants are most strongly associated with short-term improvements and long-term maintenance of physical activity. We will also investigate associations between physical activity and psychological wellbeing, specifically changes in positive and negative affect, and impaired functioning due to mental health across the intervention and follow-up period. These analyses will help identify whether higher levels of physical activity are associated with improvements in psychological wellbeing and functioning.]

**Comment 13: regarding the SPIRIT 2013 Checklist**

*METHOD Please recheck the manuscript with the SPIRIT 2013 CHECKLIST and use subheadings as appropriate for better clarification of the methods.*

Thank you, this has been revised and uploaded with the revised documents.

**Comment 14: In lines 172 to 333 (of original manuscript)**

*Explains outcome measurements. Please use Table 2 throughout the explanation of measurements instead of using almost all measurements.*

Thank you for the suggestion to remove some repetition. All of the outcomes referred to in text are now solely in Table 2. The primary outcomes are listed in the text of the methods section but detailed in the Table 2.

**Comment 15: In line 287 (of original manuscript)**

*Table 2 explains the different outcomes. Please use references for Outcome measures, Descriptions, and Measurement variables.*

Thank you for this suggestion. All of the outcomes are now referenced in the table. We have moved all of the references that were previously in-text in the methods section to Table 2.

**Comment 16: In line 363 (of original manuscript)**

*What is the meaning of the logic check? Please explain.*

Logic checks are tests used to ensure data values are consistent and make sense within the context of other data points. For example, the date at time point 1 is before the date at time point 3.

**Comment 17: In lines 364-365 (of original manuscript)**

*Please explain with appropriate reference why multiple imputations are considered.*

Repeated measures mixed models, estimated via maximum likelihood are able to handle missing data for outcome data, and imputation is not required. However, maximum likelihood does not address for missing predictor (independent covariate) data, and cases with incomplete predictor information are excluded from the analysis. To minimize data loss, we plan to consider the use of multiple imputation for predictor covariates and apply only if necessary. There are many papers a describing the use of multiple imputations with longitudinal data. We have provided one for the manuscript and have altered the manuscript to include the following:

[Patterns of missing data will be explored and multiple imputation considered where appropriate.(48)]

The added reference is:

Wijesuriya R, Moreno‐Betancur, M., Carlin, J. B., White, I. R., Quartagno, M., & Lee, K. J. . Multiple imputation for longitudinal data: A tutorial. Statistics in Medicine. 2025;44:3–4.

**Comment 18: In line 372 (of original manuscript)**

*What covariates will be used in the analysis with reference? How will you determine the covariates?*

The covariate on line 372 (of original manuscript) is the baseline measure corresponding to the outcome. For clarity we have re-phrased this sentence as below:

[Group, time, and group-by-time interactions will be included as fixed effects in the regression model. The baseline measure of the outcome will be included as a covariate to adjust for baseline differences]

**Comment 19: In line 373 (of original manuscript)**

*Please list all confounders you wish to include in the analysis.*

We have added these in the analysis section on line 406 as follows:

[The model will also be adjusted for randomisation variables and other potential confounders, such as age, surgery type, weeks since surgery and/or chemotherapy pathway (neoadjuvant or adjuvant).]

**Comment 20: regarding the methods section**
*In the methods section, there is a repetition of some information. Please remove the repetition.*

To eliminate the repetition, we have removed a lot of text (lines 289-333 of original manuscript) and put this detail into Table 2.

**Comment 21: regarding the references**

*Please follow the referencing in the main text following Plos One standard (i.e., https://journals.plos.org/plosone/article?id=10.1371/journal.pone.0327947)*

Thank you, noted, we have double checked the manuscript to ensure consistency with the Plos One standard.

**Comment 22: In line 441 (of original manuscript)**

*How suddenly a hypothesis appears. I have not seen any hypothesis mentioned in the introduction or methods. Please mention the hypothesis with an adequate explanation in the introduction and other parts as appropriate.*

Apologies for this poor choice in wording, this has been revised to “investigate if a more structured….”.

**Comment 23: In lines 451 to 453 (of original manuscript)**

*Please use reference for the following statement “As barriers to exercise are heightened for people undergoing a cancer journey, and people are at elevated risk of comorbidity following cancer treatment”.*

The appropriate reference has been added.

**Comment 24: In lines 458-460 (of original manuscript)**

*Why do you think a single centre is a limitation and reduces generalisability? Your study has several additional limitations, including the blinding of the assessor, statistician, and intervention provider, as well as the limitation of cost-saving analysis, and it also does not inform the mechanism of interventions. Please discuss these in detail, referencing them as appropriate.*

Thank you for the above points. We have revised the limitations paragraph in the discussion section of the manuscript which is pasted below.

[Limitations of this study involve the single centre design, which potentially will reduce the generalisability of the results, as usual care practices may differ between health services providers both within and outside Australia. Variations in usual care may reduce the generalisability of our estimate of the between-group differences. Although all follow-up assessments are completed by a researcher blinded to the study group, it was not possible to blind the participants or the physiotherapist delivering the intervention to their group allocation. This is a common shortcoming of studies that explore exercise interventions. Although the primary research question does not attempt to inform the mechanism of the interventions, our secondary analysis will allow us to explore factors that may modify the magnitude of between-group differences which will inform future service delivery. Overall, we feel there is high likelihood that the results will be clinically meaningful.]

In regards to the comment regarding a cost-saving analysis, we have discussed this as a team and collaborated with a health economist and potential PhD student commencing in 2026, who have an interest in utilising the EQ5D5L and the healthcare utilisation data to this effect, but as this has not yet been detailed in the study registration or ethics approval documentation so not specifically referred to in this paper.

The blinding of the statistician is detailed in lines 390-392 of the updated manuscript.

**Comment 25: In lines 451-454 (of original manuscript)**

*Please use reference for following sentences: “As barriers to exercise are heightened for people undergoing a cancer journey, and people are at elevated risk of comorbidity following cancer treatment, exercise interventions are of particular importance for improving quality of life and related function and psychological outcomes in this population.”*

A reference has been added to this statement, as per the request in *Comment 23*.

**Comment 26: regarding study strengths** *Please add a paragraph on the strength of the study.*

Thank you for this suggestion. We have added the following paragraph to the discussion:

[A major strength of this study was the co-design of the exercise intervention which took into consideration perspectives of several healthcare disciplines and, perhaps most importantly, the voices of people who have survived breast cancer. In addition, the exercise intervention was designed to be inclusive of both males and females, with any stage of breast cancer (including stage 4), who resided in either rural or metropolitan areas.]

**Comment 27: In lines 459-460 (of original manuscript)**

*Instead of the word “Confidence”, use a scientific word.*

The wording has been amended from “confident” to “high likelihood”

**Comment 28: regarding the page order** *Please correct the page orders.*

Apologies for this oversight, the page orders have been corrected.

**Reviewer #1:  Comment 1**
*A very large amount of data is to be collected on each patient so I imagine the great logistical difficulties associated. I wonder therefore if some (say 5 to 10%) of the less important variables might be dropped?*

Thank you for this comment. The potential burden of assessments has been a point of discussion among the team. As we have commenced recruitment and data collection (and the study has been registered) we are unable to change the selection of variables. However, any participant expressing concern with the burden of assessment is given the option to focus on completing the questionnaires relating to the primary outcome measurements only.

**Reviewer #1: Comment 2**

*Note a SD quoted to 2 decimal places seems a little unrealistic. I would have assumed SD = 21 with n then increasing from 105 to110 as a consequence! Clearly, the authors choice of the total sample size of N = 260 is fine.*

Noted, thank you. We have rounded the SD to a single decimal. This does not change the group numbers in sample size calculation (which are included in the study registration).

**Reviewer #2:  Comment 1 (original manuscript p12-28)**

*Please provide the minimal clinically important difference (MCID) for each outcome measure in the table.*

Where there are MCID for the breast cancer population, these have been added into Table 2.

**Reviewer #2:** **Comment 2 (original manuscript p32)**

*Given that the linear mixed effects model can appropriately handle missing data, the application of additional imputation methods may not be required. You may wish to consider whether the use of imputation is necessary in this context.*

Thank you for this comment. This has been addressed, as per *Comment 17* above.

**Reviewer #2: Comment 3 (original manuscript p31-32*)***

*As the linear mixed effects model will be employed in the data analysis, please also consider assessing the normality of the residuals. Please put it in the method part.*

Thank you for this suggestion. The following has been added to the analysis section on line 466 of the updated manuscript:

[Model fit for regression models will be performed by examining residuals (normality and constant variance). For models with repeated measures random coefficients will also be examined for normality and constant variance.]

**Reviewer #2: Comment 3 (original manuscript p*31)***

*For the sample size calculation, please place the reference citation [33] immediately following the estimated minimal clinically important difference. This would improve both the accuracy and clarity of the citation.*

This has been moved as suggested, thank you.

**Reviewer #2: Regarding the manuscript format** *Other format of manuscript, please follow the requirements of PLOS One.*

Thank you, noted, we have double checked the manuscript to ensure consistency with the Plos One standard.

We hope you find the above revisions satisfactory. If you have any queries or concerns regarding any of the above, we are very happy to discuss further.

Kind regards,

Susan Stinton
