# Supplementary material for: Evaluating the effect of the SMART intervention in people with recently diagnosed breast cancer who are being treated at a public tertiary hospital in Australia: protocol and statistical analysis plan for a single-blinded, single centre randomised controlled trial
Source: PLoS One. 2026 Jan 30;21(1):e0341423. doi: 10.1371/journal.pone.0341423 (PMC12857944; doi:10.1371/journal.pone.0341423)
Supplement: Attachment — Submitted filename: Comments-Dr. Hong Pan.docx [file pone.0341423.s008.docx]

Comments for evaluating the SMART intervention in people with recently diagnosed breast cancer: protocol and statistical analysis plan (PONE-D-25-25500)

Comments:

The author revised my suggestion, and I am very satisfied. I recommend accepting it.

Dr. Hong Pan

The Hong Kong Polytechnic University
